# Decreased plasma gelsolin fosters a fibrotic tumor microenvironment and promotes chemoradiotherapy resistance in esophageal squamous cell carcinoma

**DOI:** 10.1186/s12929-024-01078-7

**Published:** 2024-09-11

**Authors:** Chih-Hsiung Hsieh, Pei-Shiuan Ho, Wen-Lun Wang, Fu-Hsuan Shih, Chen-Tai Hong, Pei-Wen Wang, Dar-Bin Shieh, Wei-Lun Chang, Yi-Ching Wang

**Affiliations:** 1 Department of Pharmacology, College of Medicine, National Cheng Kung University, Tainan, Taiwan; 2 Department of Internal Medicine, E-Da Hospital, College of Medicine, I-Shou University, Kaohsiung, Taiwan; 3 Institute of Basic Medicine, College of Medicine, National Cheng Kung University, Tainan, Taiwan; 4 Institute of Oral Medicine, College of Medicine, National Cheng Kung University, Tainan, Taiwan; 5 Center of Applied Nanomedicine, National Cheng Kung University, Tainan, Taiwan; 6 Center for Micro/Nano Science and Technology, Advanced Optoelectronic Technology Center, Innovation Center for Advanced Medical Device Technology, National Cheng Kung University, Tainan, Taiwan; 7 Department of Internal Medicine, National Cheng Kung University Hospital, College of Medicine, National Cheng Kung University, Tainan, Taiwan

**Keywords:** Esophageal squamous cell carcinoma, Plasma gelsolin, Cancer-associated fibroblast, Tumor microenvironment, Therapeutic resistance

## Abstract

**Background:**

Stromal fibrosis is highly associated with therapeutic resistance and poor survival in esophageal squamous cell carcinoma (ESCC) patients. Low expression of plasma gelsolin (pGSN), a serum abundant protein, has been found to correlate with inflammation and fibrosis. Here, we evaluated pGSN expression in patients with different stages of cancer and therapeutic responses, and delineated the molecular mechanisms involved to gain insight into therapeutic strategies for ESCC.

**Methods:**

Circulating pGSN level in ESCC patients was determined by enzyme-linked immunosorbent assay analysis, and the tissue microarray of tumors was analyzed by immunohistochemistry staining. Cell-based studies were performed to investigate cancer behaviors and molecular mechanisms, and mouse models were used to examine the pGSN-induced tumor suppressive effects in vivo.

**Results:**

Circulating pGSN expression is distinctively decreased during ESCC progression, and low pGSN expression correlates with poor therapeutic responses and poor survival. Methylation-specific PCR analysis confirmed that decreased pGSN expression is partly attributed to the hypermethylation of the *GSN* promoter, the gene encoding pGSN. Importantly, cell-based immunoprecipitation and protein stability assays demonstrated that pGSN competes with oncogenic tenascin-C (TNC) for the binding and degradation of integrin αvβ3, revealing that decreased pGSN expression leads to the promotion of oncogenic signaling transduction in cancer cells and fibroblasts. Furthermore, overexpression of pGSN caused the attenuation of TNC expression and inactivation of cancer-associated fibroblast (CAF), thereby leading to tumor growth inhibition in mice.

**Conclusions:**

Our results demonstrated that *GSN* methylation causes decreased secretion of pGSN, leading to integrin dysregulation, oncogenic TNC activation, and CAF formation. These findings highlight the role of pGSN in therapeutic resistance and the fibrotic tumor microenvironment of ESCC.

**Supplementary Information:**

The online version contains supplementary material available at 10.1186/s12929-024-01078-7.

## Background

Esophageal cancer is one of the most aggressive cancers and the sixth leading cause of cancer mortality worldwide [1]. Histologically, 15% of cases are esophageal adenocarcinoma, and 84% of cases are esophageal squamous cell carcinoma (ESCC) in South and East Asia countries [2, 3]. In particular, an increasing trend in the incidence of ESCCs has been noted [4]. Unfortunately, about 75% of ESCC patients are diagnosed at advanced stages, in which the overall 5-year survival rate is less than 5% [5]. Currently, concomitant chemo-radiation therapy (CCRT) is used as first-line treatment for ESCC. However, many ESCC patients develop acquired therapeutic resistance and have poorer outcomes [6, 7]. Of note, stromal fibrosis has been identified to be highly associated with poor survival of ESCC patients whose tumor cells usually develop therapeutic resistance [8–10]. Previously, we reported that higher DNA methylation levels in tumor suppressor genes remarkably predicted poor responses of ESCC patients to CCRT treatment [11, 12]. Nevertheless, whether DNA methylation of tumor suppressor genes in cancer cells could drive resistance-associated fibrosis in ESCC remains to be elucidated.

Plasma gelsolin (pGSN) is the secretory isoform of cytoplasmic gelsolin (cGSN), derived from the alternative splicing of the *GSN* gene. Both pGSN and cGSN can crosslink with actin filament to regulate actin structural dynamics [13, 14], constituting the most abundant actin-binding protein in the human body. Specifically, pGSN plays an essential role in scavenging actin filaments released from dead cells into the bloodstream [15], and thus protecting normal cells against actin aggregation-induced toxicity and inflammatory responses [16]. In addition, pGSN has been identified to bind to fibronectin [17], an integrin-binding and critical extracellular matrix (ECM) protein that mediates excessive deposition of collagen during fibrosis progression [18, 19]. Moreover, decreased pGSN in the serum was discovered to induce glomerular fibrosis [20]. Therefore, it is important to understand whether pGSN correlates with tumor progression and resistance-associated fibrosis in ESCC.

In this study, we found that circulating pGSN is significantly decreased during tumor progression in ESCC patients, and low pGSN expression is associated with ESCC patients’ fibrosis, poor responses to CCRT treatment, and poor survival. Our methylation-specific PCR analysis discovered that low pGSN expression is in part attributed to DNA methylation of the *GSN* gene. Interestingly, the cell-based immunoprecipitation (IP) analysis showed a competition between pGSN and oncogenic tenascin-C (TNC) protein for binding to αvβ3 integrin, indicating that decreased pGSN expression leads to the promotion of oncogenic signaling transduction and the activation of cancer-associated fibroblast (CAF), which is validated in our in-house cohort of ESCC patients. Collectively, our findings identify pGSN as a tumor suppressor, and DNA methylation-induced low expression of pGSN leads to cancer progression, CAF activation, and CCRT resistance.

## Materials and methods

### Cell lines and culture conditions

Taiwanese ESCC cell lines CE81T and CE48T were obtained from Dr. Han-Suei Hsu (Division of Thoracic Surgery, Taipei Veterans General Hospital, Taiwan). Japanese ESCC cell line, KYSE510, was purchased from DSMZ-German Collection of Microorganisms and Cell Cultures (Braunschweig, Lower Saxony, Germany), where they were characterized by DNA-fingerprinting and isozyme detection. Taiwanese lung patient-derived CAF cells were kindly provided by Dr. Wu-Chou Su (Department of Internal Medicine, National Cheng Kung University Hospital, Taiwan). Mouse Tongue Carcinoma MTCQ1 cell line was obtained from Dr. Shu-Chun Lin (National Yang-Ming University, Taipei, Taiwan). Mouse embryo fibroblast cell line NIH/3T3 was purchased from the American Type Culture Collection (ATCC, Manassas, VA, USA). CE81T, CE48T, MTCQ1, and NIH/3T3 cells were cultured in Dulbecco's Modified Eagle Medium (DMEM, Gibco, Waltham, MA, USA) containing 10% fetal bovine serum (Gibco) and 1% penicillin/streptomycin (Gibco). KYSE510 and CAF cells were maintained in RPMI-1640 Medium (Gibco) with 10% fetal bovine serum (Gibco) and 1% penicillin/streptomycin (Gibco). All cells were cultured at 37 °C with 5% CO_2_ in air.

### Radiation treatment of cell lines

To establish the CCRT-resistant cell line, the parental cells (P) KYSE510 (KYSE510-P), CE81T (CE81T-P), and CE48T (CE48T-P) were continuously exposed to 5 Gy radiation per treatment in media containing 2 μM cisplatin (Sigma-Aldrich, Saint Louis, MO, USA). The chemoradiation-treated cells were given a chance to rest and recover until the next treatment, and received a total cumulative dose of 70 Gy radiation. The CCRT-resistant cells (R) KYSE510 (KYSE510-R), CE81T (CE81T-R), and CE48T (CE48T-R) were used for comparison studies.

### Clinical samples of ESCC patients

A total of 172 plasma samples were collected from ESCC patients in the endoscopy operation room, National Cheng Kung University Hospital, Tainan. Tumor samples used for tissue microarray were from 77 ESCC patients at the Cancer Center of National Cheng Kung University Hospital, Tainan. All patients were recruited in this study with appropriate institutional review board permission (permit #A-BR-108-069) and informed consent were obtained from the patients. The follow-up of patients was performed at 3-month intervals. The last day of the follow-up period at National Cheng Kung University Hospital was defined as March 2021. Endoscopy tumor biopsy and corresponding normal biopsy samples were collected. Patient’s response to CCRT was evaluated using endoscopic ultrasound and computed tomographic scans after completion of 36 Gy radiotherapy. The evaluation was carried out before and after CCRT therapies at the clinics. Good CCRT responders were patients with post-CCRT esophageal wall thickness < 8 mm by endoscopic ultrasound and without enlargement or newly developed distant metastatic foci on computed tomographic scan, while poor CCRT responders were patients with post-CCRT esophageal wall thickness ≥ 8 mm or with enlargement or newly developed distant metastatic foci on computed tomographic scan [21]. These patient samples were examined for GSN protein expression using enzyme-linked immunosorbent assay and immunohistochemistry staining.

### Enzyme-linked immunosorbent assay (ELISA)

The secretion level of human pGSN was detected by Gelsolin (Plasma) (Soluble) (Human) ELISA Kit according to the manufacturer’s instructions (AVISCERA BIOSCIENCE, Santa Clara, CA, USA). Briefly, 10,000 dilutions of plasma samples was prepared and analyzed concurrently with a range of known pGSN standards. The optical absorbance at 450 nm was measured.

### The cancer genome atlas (TCGA) and genotype-tissue expression (GTEx) datasets analysis

The gene expression data of 62 ESCC samples from TCGA and 649 normal esophageal tissue samples from GTEx were derived from UCSC Xena (https://xena.ucsc.edu/). The HTseq-Counts were converted to FPKM (Fragments Per Kilobase per Million mapped reads) normalized data. The Log2 transformation of FPKM + 1 was used to present the differential expression of the *GSN* gene in normal and tumor samples.

### Expression vectors and transfection

The entire coding region of *pGSN* PCR product was restricted using *Bamh I* and *XhoI* enzymes and then subcloned in frame into the pcDNA3.1/myc-His A vector. The pGSN-expressing vector was introduced into target cells using Turbofect reagent (Invitrogen, Waltham, MA, USA) according to manufacturer instructions. After 24 h incubation, the transfected cells were harvested for further assays. The plasmids used in the study are listed in Table S1.

### Conditioned media (CM) collection, protein extraction, and western blot analysis

CM were harvested from the supernatant of cancer cells after transfection with pGSN-expressing vector for 24 h. 0.1% v/v protease inhibitors cocktail (Sigma-Aldrich) and 0.1% v/v 1 M dithiothreitol were added to the collected CM (pGSN-CM), and centrifuged (Z 216 MK, HERMLE, Germany) at 13,200 rpm at 4 °C for 15 min. The supernatant was collected for further assays.

The transfected cells were lysed on ice using the RIPA buffer containing protease inhibitors cocktail (Sigma-Aldrich), and the lysates were then centrifuged at 13,200 rpm for 15 min. An equal amount (50 µg) of protein extract was separated on a 8% SDS-PAGE and transferred onto a polyvinyl difluoride membrane. The membranes were blocked with 5% skim milk in Tris-buffered saline with 0.1% Tween-20 for 1 h at room temperature and subsequently incubated with primary antibodies at 4 °C overnight, followed by incubation with horseradish peroxidase-conjugated secondary antibodies. The antibody conditions are described in Table S2.

### RNA extraction and quantitative reverse transcription polymerase chain reaction (RT-qPCR)

Total RNA was extracted using TRIzol reagent (Invitrogen), and purified RNA was reversely transcribed into cDNA using the High Capacity cDNA Reverse Transcription Kit (Applied Biosystems, Carlsbad, CA, USA). RT-qPCR was performed with SYBR Green Master Mix (Invitrogen) using the StepOnePlus™ Real-Time PCR system (Applied Biosystems). Expression levels were normalized with internal control β-actin. The primer sequences are listed in Table S3.

### DNA demethylation treatment

ESCC cells were seeded (1 × 10^6^ cells) in 10 cm dish and were treated with 100 µmol/L 5-aza-2’-deoxycitidine (5-aza, Sigma-Aldrich) or nutlin-3 (Selleck Chemicals, Houston, TX, USA) for 3 days, with replacement of fresh medium containing demethylation reagent 5-aza or nutlin-3 every 24 h. After treatment, total genomic DNA, RNA, and protein were extracted for the analysis of *pGSN* gene methylation status, and mRNA and protein expression.

### DNA bisulfite conversion and methylation-specific polymerase chain reaction (MSP)

Genomic DNA was extracted using the Quick-DNA™ Miniprep Plus Kit (Zymo Research, Orange, CA, USA), and then an equal amount of DNA samples was treated with EZ DNA methylation-Gold kit (Zymo Research). The methylation level was determined by PCR analysis using the primers specific for either methylated (M) or unmethylated (U) DNA. Primers are listed in Table S3.

### Immunohistochemistry (IHC) staining

All slides were dewaxed with xylene and antigens were retrieved by incubating the slides for 10 min at 100 °C. Then IHC staining was performed using Novolink Max Polymer kit (Leica Biosystems, Wetzlar, Germany) according to the manufacturer’s instructions. Evaluation of IHC was conducted blindly without knowledge of the clinical and pathologic characteristics of the patients. The staining was scored with brown-staining intensity and expression area percentage translated into a four-tier system, including strong positive (100–75%) as 4, moderate (75–50%) as 3, weak (50–25%) as 2 or negative (25–0%) as 1. Tumor spots of each patient on tissue microarray were scored and then averaged as the score of expression level. The antibody conditions are listed and described in Table S2.

### Cell-based IP

ESCC cells were seeded (3 × 10^6^ cells/well) in 10 cm dish. To perform IP analysis of cell surface protein, cells were washed with PBS and incubated with medium containing normal mouse IgG or integrin αvβ3 antibody on a shaker at 4 °C for 1 h. After hybridization, cells were rinsed with ice-cold PBS twice and lysed with 1 × IP buffer (50 mM Tris–HCl, pH 7.5, 150 mM NaCl, 20 mM α-glycerol-phosphate, 1% NP-40, 5 mM EDTA, 0.01% protease inhibitor cocktail) at 4 °C for 15 min. Cell lysate was sonicated, and centrifuged at 13,200 rpm at 4 °C for 15 min to isolate the supernatant. The protein extracts were quantified (500 μg) and incubated with 15 μL Protein G/Protein A agarose beads (Calbiochem, San Diego, CA, USA) at 4 °C overnight. Complexes were then washed with 1 × IP buffer 3 times. Proteins were eluted by boiling in 2 × SDS loading buffer, separated by 8% SDS-PAGE, then blotted with αvβ3 integrin, pGSN, or TNC antibodies. The antibodies conditions are listed and described in Table S2.

### Cycloheximide (CHX) chase assay

For CHX chase assay, CE81T-R and 3T3 cells were treated with CM from empty vector (EV) control or pGSN-overexpressing (OE) ESCC cells along with cycloheximide (CHX, 20 μg/mL), a potent inhibitor of protein synthesis. Protein lysates were harvested at the indicated times and analyzed by immunoblotting analysis.

### Immunofluorescence (IF)

For immunofluorescence staining, Opal stain kit (PerkinElmer, Waltham, MA, USA) was utilized. Cells were fixed with 4% formaldehyde and then antigen retrieval was performed with citrate buffer (pH 6.0) at 100 °C for 20 min. After blocking, the slides were incubated with primary antibody of p-FAK or p-paxillin, followed by incubation with secondary antibody polymer HRP and subsequently with Opal fluorophore for 10 min at room temperature. Finally, DAPI was applied for nuclear staining, and images were visualized using a fluorescence microscope (Olympus, Tokyo, Japan). The antibody conditions are listed in Table S2.

### Collagen gel contraction assay

Collagen gel matrices were prepared by adding 250 μL of a neutralized collagen (Vitrogen, Angiotech, Palo Alto, CA, USA) solution [8 mL collagen (3 mg/mL), 1 mL 10X DMEM, 1 mL 200 mmol/L HEPES, pH 8.5], to a 24-well tissue culture well. After incubating CAFs on the restrained gels, gels were released and their two longest diameters were measured at the indicated time points. Gel size was defined as the sum of the two gel diameters and gel contraction expressed as a percentage of the original gel size.

### Animal studies

All animal experiments were performed in compliance with institutional guidelines for use and care of animals (permit #109,060). To investigate the role of pGSN in tumor growth, 5–6-week-old BALB/c nude mice were subcutaneously implanted with 5 × 10^6^ CE81T-R cells. To evaluate the anti-CAF effects of pGSN, 5–6-week-old BALB/c nude mice were subcutaneously co-implanted with 5 × 10^6^ CE81T-R and 5 × 10^5^ 3T3 fibroblasts.

Mice were weighed, and the volumes of the xenografts were measured and quantified during experiment. At the endpoint of the experiments, tumor tissues were excised and then subjected to RNA extraction or fixed with 4% formaldehyde (Sigma-Aldrich).

### Statistical analysis

The statistical analyses of pGSN expression level, overall survival, and cancer risk factors were performed using Statistical Package for the Social Sciences version 26.0 (SPSS Inc., Chicago, IL, USA). The chi-square test and multivariate logistic regression analyses were conducted. Correlations were examined using Pearson’s correlation test. Overall survival curves were calculated according to the Kaplan–Meier method using the log-rank test. Three independent experiments for cell studies and six mice per group for animal studies were analyzed. Two-tailed Student’s t-test and one-way ANOVA test were used in cell and animal studies. Data represented mean ± s.e.m. The levels of statistical significance were expressed as p-values, *p < 0.05; **p < 0.01; ***p < 0.001; ****p < 0.0001; ns: non-significant.

## Results

### Low pGSN level correlates with *cancer* progression and poor prognosis in ESCC patients

**Fig. 1:**
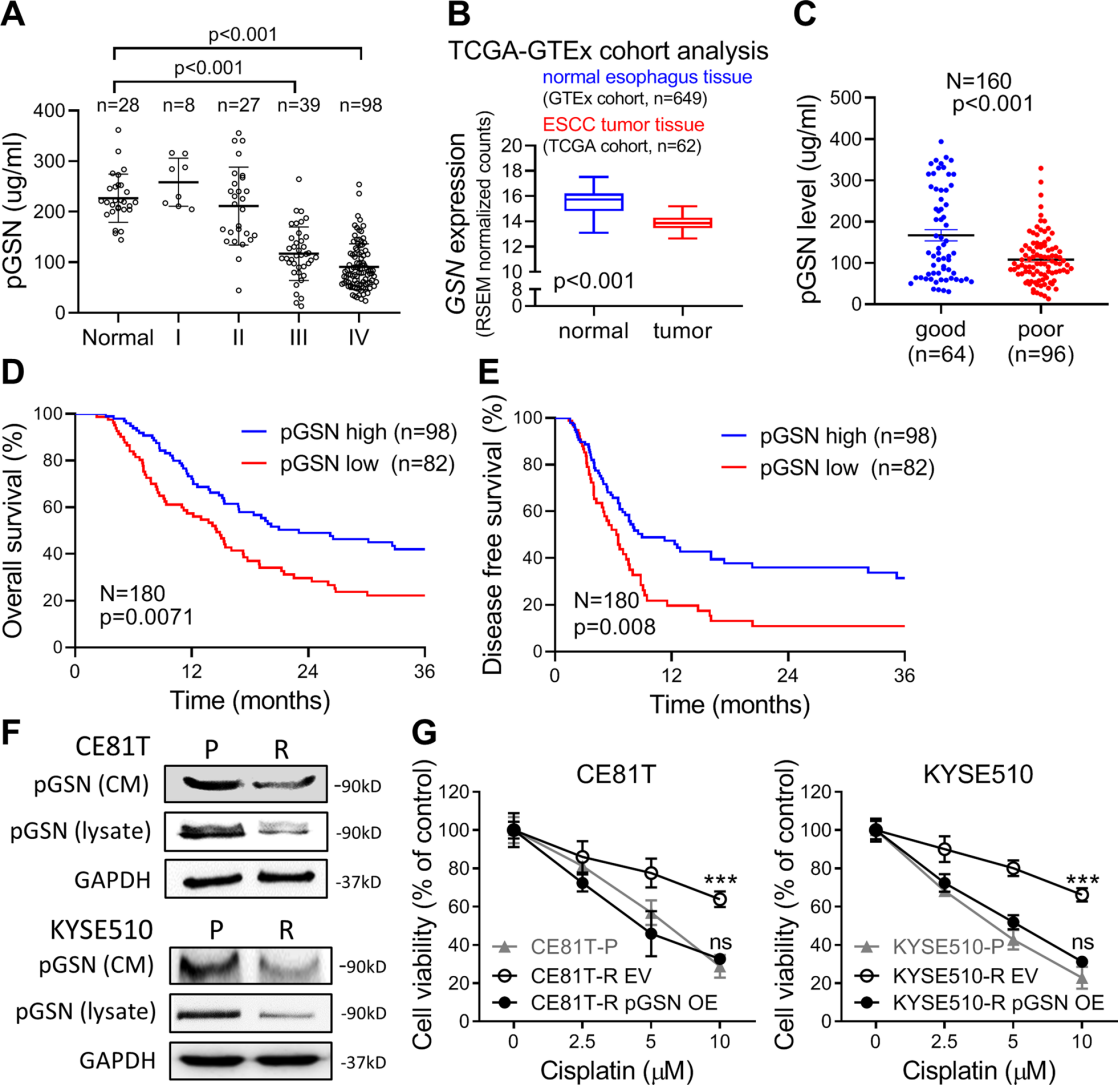
Low pGSN level correlates with cancer progression and poor prognosis in ESCC patients. **A** ELISA analysis of circulating pGSN in ESCC patients (N = 172) stratified by different stages. A total of 28 normal individuals were included as a control group. **B** Quantitative difference of *GSN* expression in the GTEx (normal) and TCGA (tumor) datasets. **C** ELISA analysis of circulating pGSN in ESCC patients with good CCRT response (n = 64) or poor CCRT response (n = 96). **D** and **E** Kaplan–Meier plots of overall (**D**) and progression-free survival. (**E**) Survival analysis based on the circulating pGSN expression. The optimal cutoff level was set using Fisher’s exact test. **F** Basal levels of pGSN expression in cell lysate and CM of the resistant (R) or parental (P) cells were examined by Western blot analysis. GAPDH was used as an internal control. **G** MTT assay of resistant and parental ESCC cells treated with various concentrations of cisplatin for 72 h. Data represents mean ± s.e.m. ns: non-significant; *p < 0.05; **p < 0.01; ***p < 0.001

To understand the tumorigenic role of pGSN in ESCC, we used ELISA to evaluate the circulating pGSN level in plasma samples and its correlation with the survival of ESCC patients. The results demonstrated that pGSN level was significantly lower in patients with advanced-stage (stage III and IV) cancer as compared to early-stage (stage I and II) or normal plasma. Notably, there was no difference in the GSN level between early-stage and normal plasma groups (Fig. 1A and Fig. S1A). Moreover, chi-square analysis revealed that lower pGSN level was significantly associated with tumor staging and poor outcome (Table S4). Consistently, the analysis of TCGA (TCGA-ESCC) and GTEx normal esophagus tissue database showed that the mRNA level of *GSN* was lower in ESCC tumors than that in normal tissues (Fig. 1B). Of note, we observed that *pGSN* mRNA expression was significantly decreased in patients with poor response to CCRT treatment than in good responders (Fig. 1C). In addition, Kaplan–Meier survival analysis indicated that low pGSN level in patients correlated with poor overall survival and poor disease-free survival (Fig. 1D and E). We further performed the multivariate Cox regression to demonstrate the prognostic effect with low pGSN circulation levels. Table 1 showed that low pGSN level was potentially an independent prognostic factor in ESCC patients even after adjusting for other variables including tumor stages and metastasis status by the multivariate analysis (pGSN: hazard ratio, HR, 1.626; 95% confidence interval CI, 0.982–2.694; *p* = 0.059). These results suggested that pGSN plays a tumor-suppressive role and serves as a potential non-invasive biomarker to predict prognosis in ESCC.

**Table 1:** Cox regression analysis of risk factors for cancer-related death in ESCC patients

Characteristics		Univariate analysis		Multivariate analysis	
		HR^a^ (95% CI^a^)	*p* value^b^	HR^a^ (95% CI^a^)	*p* value^b^
pGSN expression level	High	1	**0.007**	1	0.059
	Low	1.877 (1.190–2.961)		1.626 (0.982–2.694)	
Age	≥ 55	1	0.283	1	0.890
	< 55	0.988 (0.967–1.010)		0.969 (0.619–1.517)	
Gender	Female	1	0.237	1	0.317
	Male	1.842 (0.670–5.069)		1.799 (0.570–5.681)	
Alcohol	No	1	0.569	1	0.712
	Yes	1.223 (0.612–2.444)		0.848 (0.355–2.029)	
Smoker	No	1	0.277	1	0.202
	Yes	1.446 (0.744–2.809)		1.547 (0.792–3.022)	
Stage	I, II, III	1	**0.021**	1	0.172
	IV	1.715 (1.086–2.710)		1.432 (0.856–2.398)	

^a^HR: Hazard ratio; CI: Confidence interval

^b^Bold values indicate statistical significance (*p* < 0.05)

To further investigate the role of pGSN in therapeutic resistance, we established CCRT-resistant cell lines and found that the resistant cells (R) expressed lower pGSN protein in CM and showed lower *pGSN* mRNA level than in the parental cells (P) (Figs. 1F, S1B and C). Notably, the MTT assay demonstrated that pGSN overexpression reduced the viability of resistant cells (R pGSN OE) to a level similar to that in the P cells under cisplatin treatment (Fig. 1G), indicating that the therapeutic resistance of cancer cells may result from lower expression of pGSN.

### Overexpression of pGSN reduces the in vitro oncogenicity of ESCC cells

**Fig. 2:**
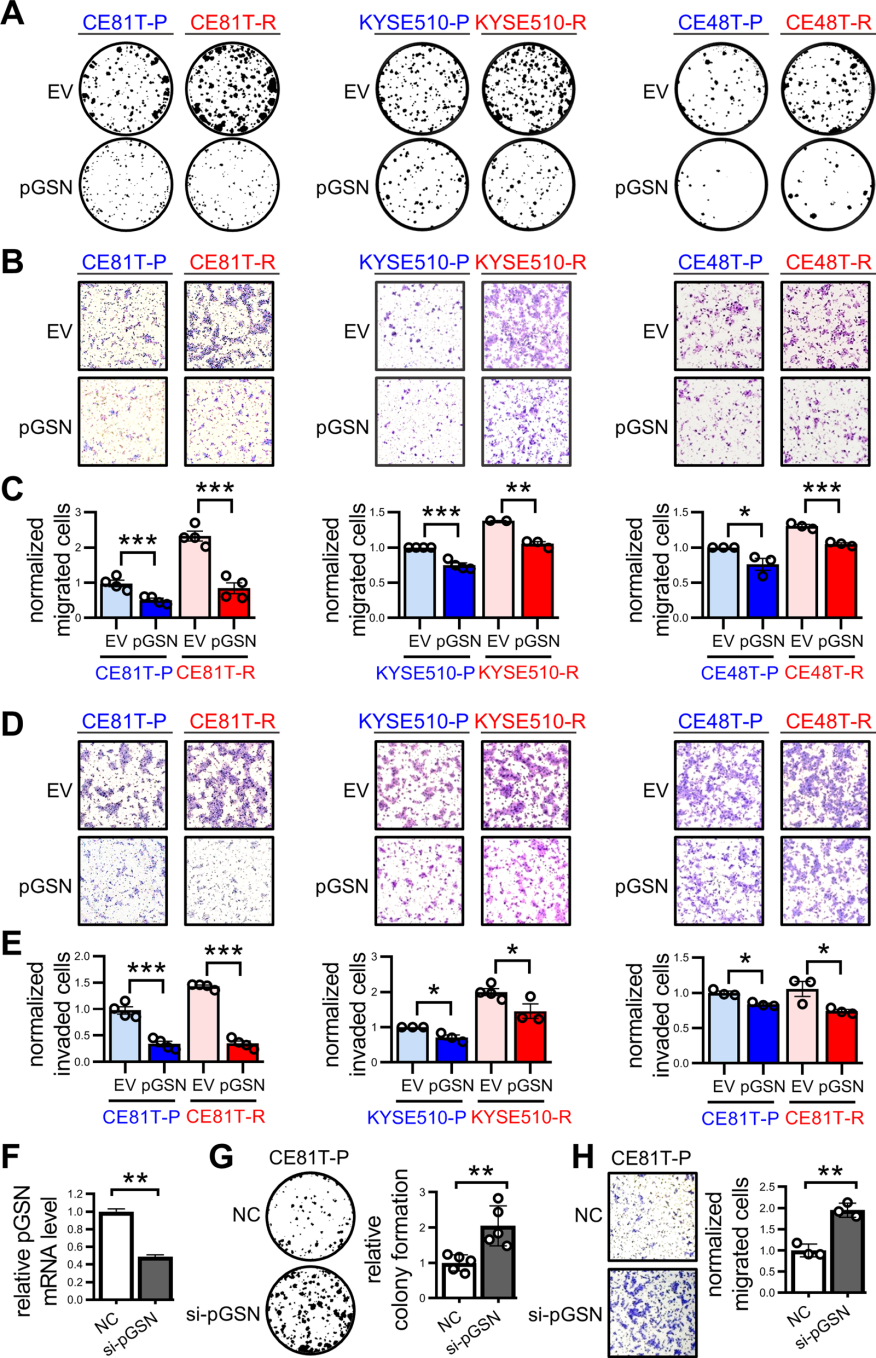
Overexpression of pGSN reduces the growth and migration of ESCC cells in vitro. **A** Colony formation assay of ESCC parental (P) or resistant (R) cells manipulated with pGSN expression. The colonies were stained on day 8 after seeding. **B** and **C** Transwell migration assay of ESCC parental (P) or resistant (R) cells manipulated with pGSN expression. The migrated cells were photographed (**B**) and quantified (**C**) at 16 h. **D** and **E** Transwell invasion assay of ESCC parental (P) or resistant (R) cells manipulated with pGSN expression. The invaded cells were photographed (**D**) and quantified (**E**) at 20 h. **F** RT-qPCR analysis of pGSN expression after knockdown of pGSN in CE81T-P cells. NC: negative control. **G** Colony formation assay of CE81T-P cells manipulated with pGSN expression. The colonies were stained on day 8 after seeding. **H** Transwell migration assay of CE81T-P cells manipulated with pGSN expression. The migrated cells were photographed and quantified at 16 h. Data represents mean ± s.e.m. ns: non-significant; *, p < 0.05; **, p < 0.01; ***, p < 0.001

Next, to verify whether pGSN could affect cancer cell behaviors in ESCC, we conducted colony formation, trans-well migration, and invasion assays for cancer cells manipulated with pGSN overexpression (Fig. S2A). As shown in Fig. 2A, the resistant cells exhibited more aggressive colony-forming ability than the parental cells. Interestingly, pGSN overexpression in both parental and resistant cells could reduce the number and size of cancer cell colonies, indicating that pGSN overexpression could attenuate cell proliferation in ESCC cells. Additionally, the trans-well migration assay showed that cell migration ability was increased in resistant cells as compared to the parental cells, and cell migration ability of both parental and resistant cells was decreased after pGSN overexpression (Fig. 2B and C). Moreover, the invasive capacity of ESCC cells was also attenuated after overexpression of pGSN (Fig. 2D and E). By contrast, pGSN knockdown enhanced not only the number and size of cancer cell colonies but also the migration ability of ESCC cells (Fig. 2F–H). The reconstitution experiment by knockdown of pGSN in ESCC cells overexpressing pGSN reversed the anti-proliferation effect of pGSN (Fig. S2B). Collectively, these results revealed that low pGSN expression in the resistant cells could result in more aggressive proliferation, migration, and invasion abilities. In addition, pGSN overexpression reduced the oncogenicity of both parental and resistant ESCC cells.

### Low pGSN expression is partly attributed to DNA hypermethylation of its promoter

**Fig. 3:**
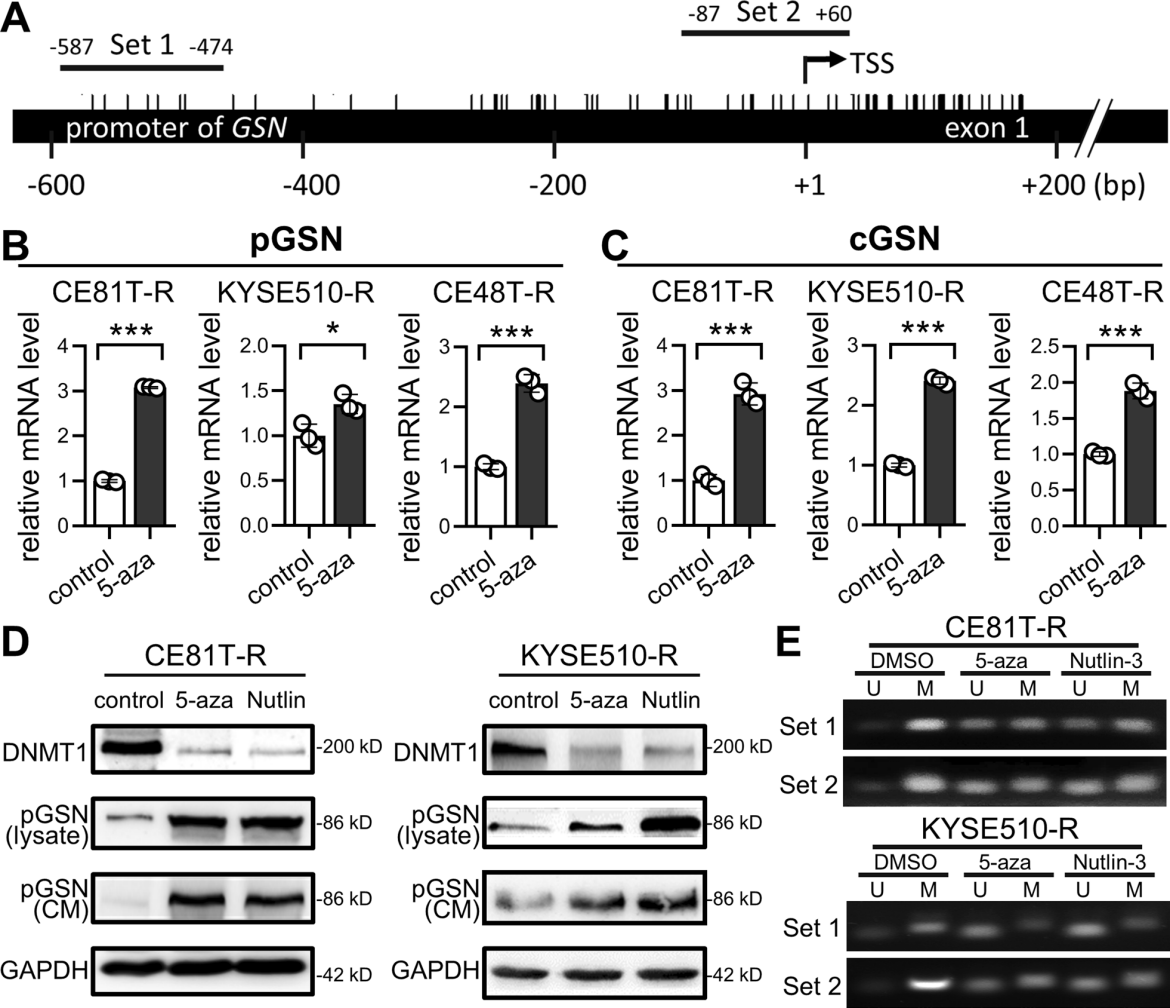
Promoter hypermethylation results in low pGSN expression in ESCC cells. **A** The promoter map of *GSN* gene. Each bar marked on the DNA strand indicates a CpG site. Primer sets for MSP analysis are labeled as Set 1 and Set 2. **B** and **C** RT-qPCR analysis of *pGSN* (**B**) and *cGSN* (**C**) mRNA expression. *β-actin* was used as an internal control. **D,** Immunoblotting of DNMT1 and pGSN in ESCC cells treated with demethylation agents 5-aza or Nutlin-3. GAPDH was used as an internal control. **E** MSP results demonstrated that demethylation agents (5-aza or Nutlin-3) reduced the methylation of the *GSN* promoter, as evidenced by an increase in U products. Data represents mean ± s.e.m. ns: non-significant; *p < 0.05; **p < 0.01; ***p < 0.001

Since aberrant epigenetic alterations can lead to silencing of tumor-suppressive genes, and pGSN expression is down-regulated in ESCC, we hypothesized that lower pGSN expression in ESCC could be attributed to DNA methylation of gene promoter. First, we identified many CpG sites on *GSN* gene by DNA sequence mapping (Fig. 3A). Following, we conducted RT-qPCR analysis to investigate whether *pGSN* mRNA expression was increased after treatment with demethylation agent 5-aza. As shown in Fig. 3B, the pGSN expression in ESCC was up-regulated after 5-aza demethylation treatment. Interestingly, mRNA expression of *cGSN*, an alternative splicing variant of *GSN* gene, was also increased in the cells treated with demethylation agent (Fig. 3C). Moreover, we found that the resistant cells showed lower mRNA levels of both pGSN and cGSN (Figs. S1A and S3A), indicating that resistance-associated low pGSN expression could be associated with promoter methylation of *GSN* gene locus. In agreement with the results of RT-qPCR analysis, the protein expressions of pGSN in ESCC cell lysate or CM were increased after demethylation treatment (5-aza or Nutlin-3 [22]) (Figs. 3D and S3B). To confirm the effect of demethylation treatments on *GSN* promoter, we used M and U primers to measure the methylation level of two CpG regions (sets 1 and 2) as shown in Fig. 3A. Of note, the results of methylation-specific PCR demonstrated that the two CpG regions on *GSN* promoter were hypermethylated (Figs. 3E and S3C). Importantly, the methylation levels of these CpG regions were both obviously decreased after demethylation treatments, indicating that low pGSN expression in ESCC is in part attributed to promoter hypermethylation of the *GSN* gene.

### pGSN inversely correlates with DNA methyltransferases (DNMTs) and cancer-associated fibroblast markers in clinical ESCC tissues

**Fig. 4:**
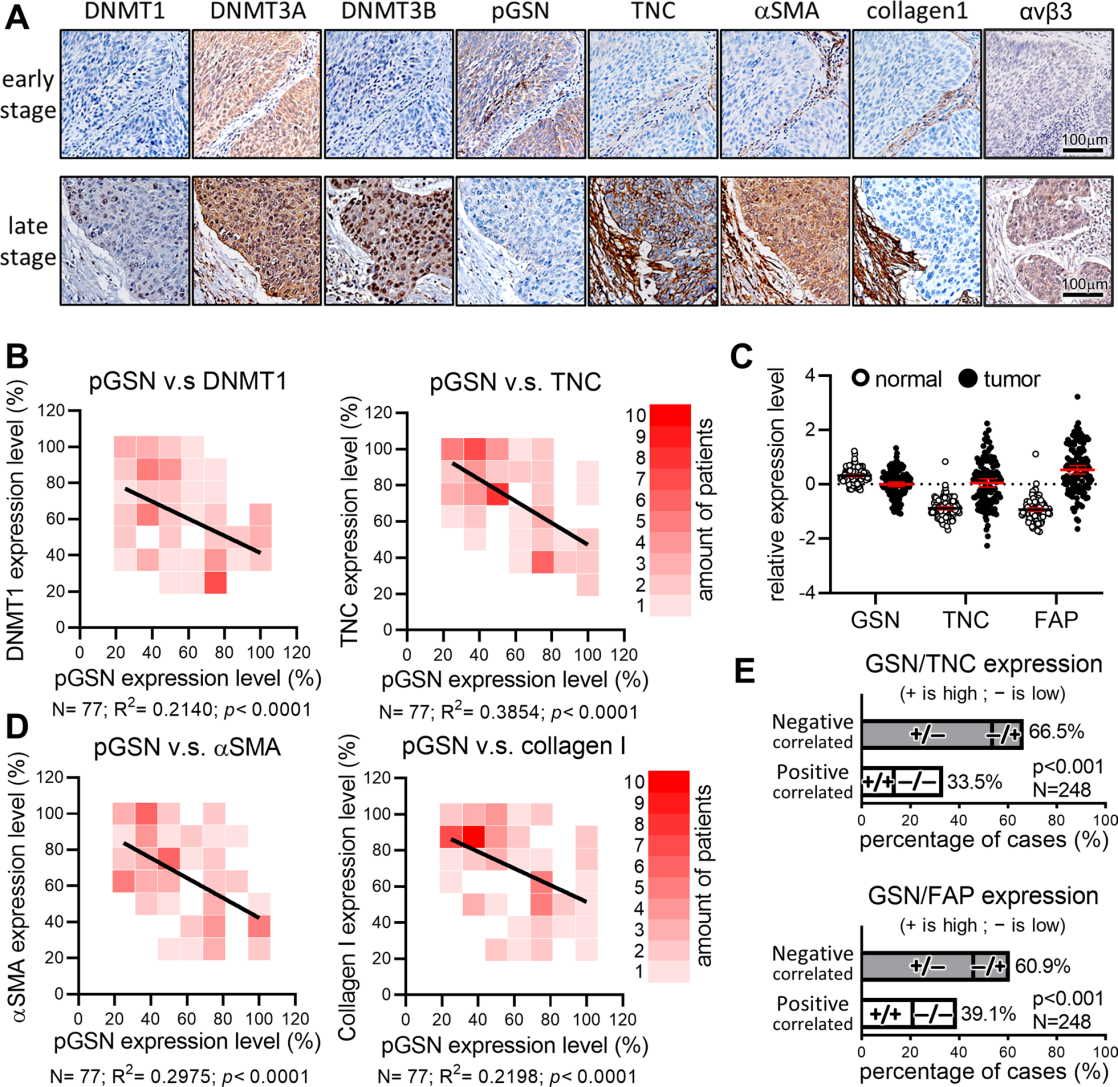
pGSN inversely correlates with the expression of DNMTs and CAF markers. **A** Representative IHC staining images of DNMT1, DNMT3A, DNMT3B, pGSN, TNC, αSMA, collagen 1, and integrin αvβ3 expression in early and late-stage ESCC patients. **B** Correlation analysis of pGSN with DNMT1 (*left*) and TNC (*right*). **C** Protein levels of GSN, TNC, and FAP in the ESCC proteomic mass spectrum dataset (24). **D** Correlation analysis of pGSN with αSMA (*left*) or collagen 1 (*right*). **E** Proteomics revealed that most ESCC patients show an inverse expression of pGSN with TNC (*upper*) or FAP (*bottom*)

So far, our results suggested that pGSN may play a tumor suppressive role in ESCC, and reduction of pGSN expression during tumor progression could result from promoter methylation. Accordingly, we performed IHC analysis to examine the expression of DNMTs in ESCC patient’s tumor tissues. As shown in Fig. 4A, the IHC results demonstrated that the expression of DNMTs was notably increased in late-stage patients as compared to early-stage patients. In addition, quantitative analysis of IHC staining revealed a significant inverse correlation between pGSN and DNMTs (Figs. 4B and S4A).

Since pGSN is a secreted protein, we examined the roles of pGSN in extracellular compartment. We also evaluated the protein expression of TNC, an oncogenic ECM protein promoting ESCC stemness [23]. The quantitative IHC analysis of patient tumor tissues revealed a notable inverse correlation between pGSN and TNC (Fig. 4A and B). In addition, by mining the published proteomic dataset [24], we confirmed that the GSN expression level was decreased whereas the TNC expression level was increased in tumors as compared to normal tissues (Fig. 4C). Of note, the proteomics analysis also showed that tumors expressing higher level of fibroblast activation protein (FAP), a CAF marker, positively correlated with ESCC chemoradiotherapeutic resistance [25] (Fig. 4C). Importantly, the IHC analysis demonstrated that CAF markers, α smooth muscle actin (αSMA) and collagen I as well as integrin αvβ3, a transmembrane molecular for ECM signaling, were highly expressed in late-stage tumor tissues (Fig. 4A). αSMA and collagen I were inversely correlated with pGSN (Fig. 4D), suggesting that pGSN may play a role in resistance-associated fibrosis of ESCC. Consistent with our IHC analysis, the proteomic dataset also showed that pGSN was inversely correlated with oncogenic TNC and CAF markers (Fig. 4E). The up-regulation of integrin αv in ESCC and its trend of positive correlation with poor survival were validated in the published proteomic dataset [24] (Fig. S4B and C). Taken together, these results indicated that extracellular pGSN is involved in ECM regulation and activation of CAFs.

### Extracellular pGSN binds to integrin αvβ3, thus suppressing the oncogenic TNC pathway

Integrins are widely known transmembrane receptors for ECM proteins and they also transduce extracellular signaling into the cell [26]. In addition, many studies have reported that TNC can enhance its own synthesis via a positive feedback loop through integrin pathways [27–29]. Additionally, we utilized computational tool AlphaFold 3.0 to predict the potential interaction between pGSN and TNC with integrin αvβ3. We found that TNC formed a tight heteromer complex with integrin αvβ3 (Fig. S5A), while the complex structure seemed to loosen upon binding with pGSN (Fig. S5B). Moreover, the docking scores suggested that GSN may interact with the integrin through amino acids TYR208 and ASP248 (Fig. S5C), which were the same residues bound with TNC (Fig. S5D). The AlphaFold2 prediction and Chimera software analysis suggested that GSN may compete with TNC to interact with integrin αvβ3. Therefore, we hypothesized that pGSN may disrupt the oncogenic TNC-integrin feedback loop, thereby eliciting tumor suppressive effects.

**Fig. 5:**
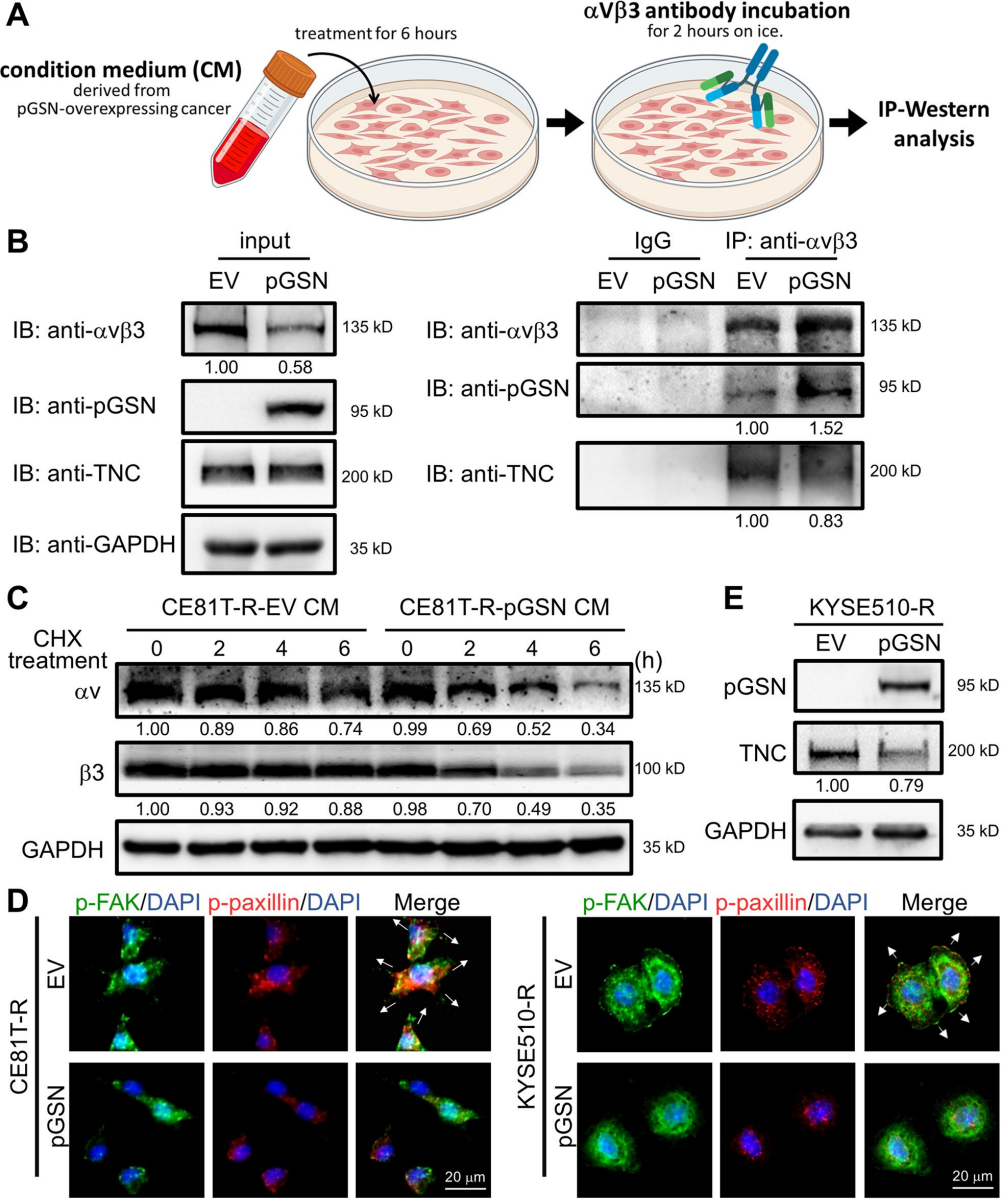
pGSN competes with TNC for binding to integrin αvβ3. **A** Schematic of cell-based IP analysis. **B** Western blot analysis of membrane-anchored integrin IP discovered an integrin-binding competition between pGSN and TNC in ESCC. GAPDH was used as an internal control. **C** Western blot analysis of CHX chase assay to investigate the protein stability of integrin αvβ3. GAPDH was used as an internal control. **D** Integrin downstream signaling p-FAK and p-paxillin was examined by IF staining. **E** Western blot analysis of TNC expression in pGSN-overexpressing cells. GAPDH was used as an internal control

Among the integrin protein family, αvβ3 has been identified to be up-regulated in ESCC and positively correlated with poor survival of ESCC patients [30]. Accordingly, we performed cell-based IP analysis to validate whether pGSN could bind to integrin αvβ3 and thus reduce the binding of TNC to αvβ3 (Fig. 5A). The results showed that treatment with pGSN-CM derived from cancer cells overexpressing pGSN increased the interaction of αvβ3 with pGSN (Fig. 5B). Strikingly, the binding of TNC to αvβ3 was reduced by pGSN-overexpressing CM treatment (Fig. 5B), suggesting that pGSN can compete with TNC for binding to integrin. Interestingly, as shown in Western blotting of input samples, we observed that pGSN overexpression reduced the protein expression of integrin αvβ3 (Fig. 5B). Next, we performed CHX chase assay and found that pGSN-overexpressing CM treatment reduced protein stability of integrin αv and β3 (Fig. 5C). Subsequently, IF staining demonstrated that integrin downstream signaling (phosphorylation of FAK and paxillin) was suppressed, and ESCC cell shrinkage was induced after pGSN-overexpressing CM treatment (lower, Figs. 5D and S5E). Furthermore, we examined whether TNC positive feedback loop could be disrupted, and the result showed that TNC protein expression was decreased after pGSN overexpression (Fig. 5E). Altogether, these results demonstrated that extracellular pGSN competes with TNC for binding to integrin αvβ3, and thus disrupting the oncogenic TNC positive feedback loop.

### Cancer-derived extracellular pGSN induces instability of fibroblast integrin to suppress CAF activation

**Fig. 6:**
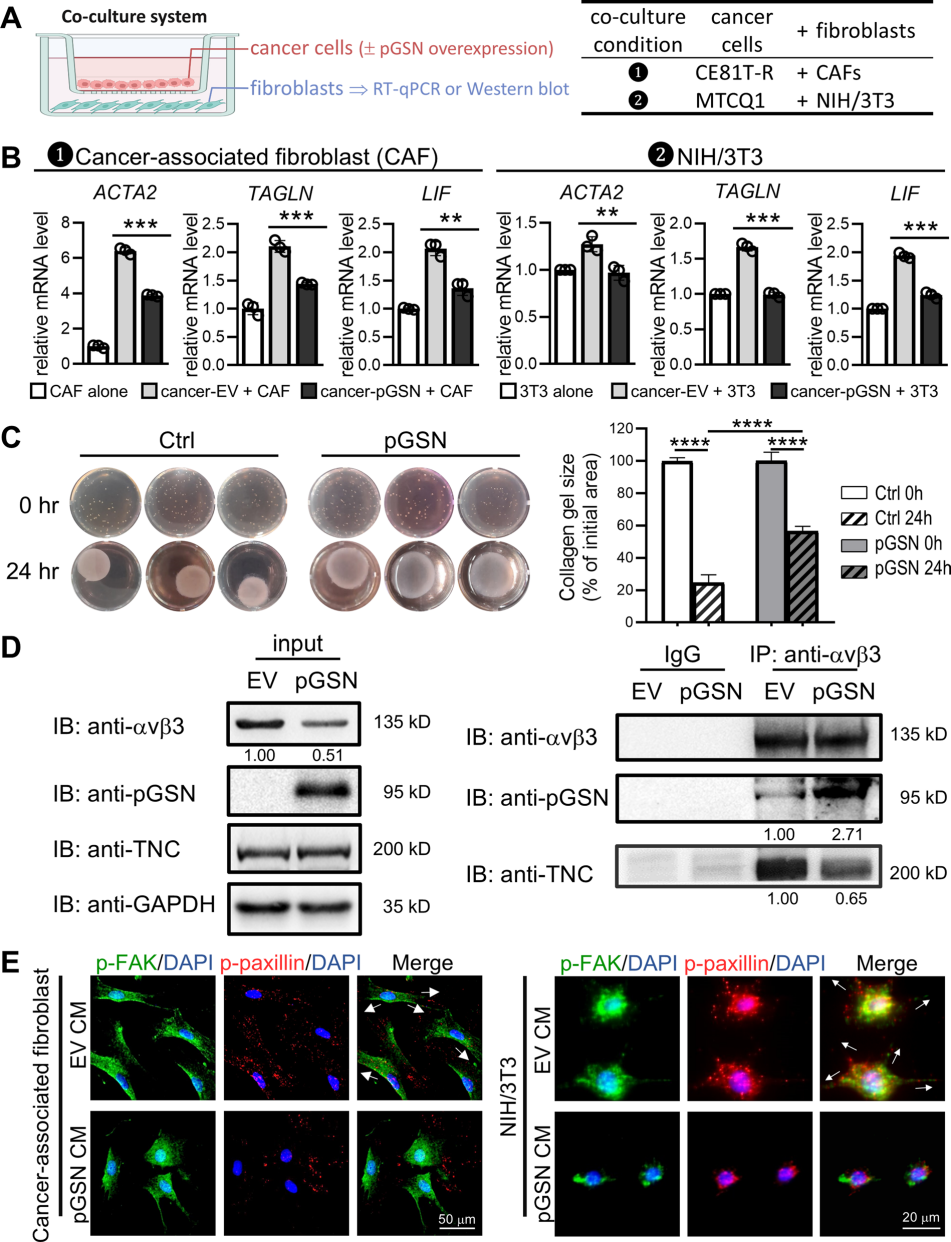
pGSN attenuates CAF activation by inhibiting integrin pathway. **A** Schematic of a co-culture system comprising of cancer cells and fibroblasts *(left*). Two conditions of the co-culture are listed (*right*). **B** CAF markers (*ACTA2*, *TAGLN*, and *LIF*) were analyzed by RT-qPCR. *β-actin* was used as an internal control. **C,** The collagen contraction analysis demonstrated an attenuation of extracellular pGSN on collagen contraction induced by CAFs. **D** Western blot analysis of membrane-anchored integrin IP discovered an integrin-binding competition between pGSN and TNC in fibroblasts. GAPDH was used as an internal control. **E** Integrin downstream signaling p-FAK and p-paxillin was examined by IF staining. Data represents mean ± s.e.m. ns: non-significant; *p < 0.05; **p < 0.01; ***p < 0.001

In the IHC analysis of patient’s tumor tissues (Fig. 4A), we discovered that CAF markers (αSMA and collagen I) were inversely correlated with pGSN. Therefore, we conducted a co-culture system of cancer cells and fibroblasts to investigate whether extracellular pGSN may be involved in CAF activation (Fig. 6A). As shown in Fig. 6B, the RT-qPCR analysis indicated that mRNA expression of CAF markers (*ACTA2, TAGLN,* and *LIF*) was increased when fibroblasts were educated by cancer cells (*gray bars*, cancer-EV + CAF). Importantly, the cancer-induced activation of CAF markers was significantly decreased when fibroblasts were educated by pGSN-overexpressing cancer cells (*black bars*, cancer-pGSN + CAF, Fig. 6B). Similar results were obtained in the co-culture system of mouse MTCQ1 oral cancer cells and mouse NIH/3T3 fibroblasts (Fig. 6B, *right*), suggesting that pGSN attenuates fibroblast activation in the cancer cells/fibroblasts co-culture system. In addition, the collagen contraction experiment showed a strong contraction when CAFs were cultured on collagen gel (Fig. 6C, *left* and *right*). Importantly, the addition of extracellular pGSN attenuated the contraction induced by CAF (Fig. 6C, *middle* and *right*). These results supported the involvement of extracellular pGSN in regulating the ECM and activating CAFs.

Accumulated studies have shown that integrin proteins are involved in the activation and maintenance of CAF [31]. We found that extracellular pGSN reduced the protein stability of integrin αvβ3. Thus, we hypothesized that pGSN-mediated inhibition of CAF activation may be attributed to pGSN-induced suppression of integrin αvβ3 pathway. Interestingly, as shown in Fig. S6, the CHX chase assay demonstrated that the protein stability of integrin αvβ3, which is expressed on fibroblast membrane, was decreased by the treatment of pGSN-overexpressing CM derived from cancer cells. Since extracellular TNC was reported to induce phenotypic changes in fibroblasts toward activated CAF via integrin signaling transduction [29], we further performed cell-based co-IP analysis to validate whether pGSN-induced suppression of CAF is associated with integrin-binding competition between pGSN and TNC. The results showed that the binding of TNC to fibroblast integrin αvβ3 was drastically decreased after pGSN-overexpressing CM treatment (Fig. 6D). In addition, IF analysis demonstrated that integrin downstream signaling proteins, p-FAK and p-paxillin, were suppressed and fibroblast shrinkage was notably observed after pGSN-overexpressing CM treatment (Fig. 6E). Collectively, these results revealed that cancer-derived extracellular pGSN competes with TNC for binding to fibroblast integrin αvβ3, leading to inhibition of the integrin pathway and suppression of CAF activation.

### pGSN significantly suppresses tumor growth by reducing TNC expression and CAF activation in vivo

To investigate whether pGSN plays as a tumor suppressor in vivo, xenograft animal models of CE81T-R (EV) and CE81T-R (pGSN) were established. We found that tumor growth and tumor size were significantly reduced when pGSN was overexpressed in cancer cells (Fig. S7A-C). Particularly, ELISA analysis confirmed that circulating pGSN level was higher in the endpoint serum of pGSN overexpression group than that of EV group (Fig. S7D). In addition, the body weight of mice was not affected by pGSN manipulation (Fig. S7E).

**Fig. 7:**
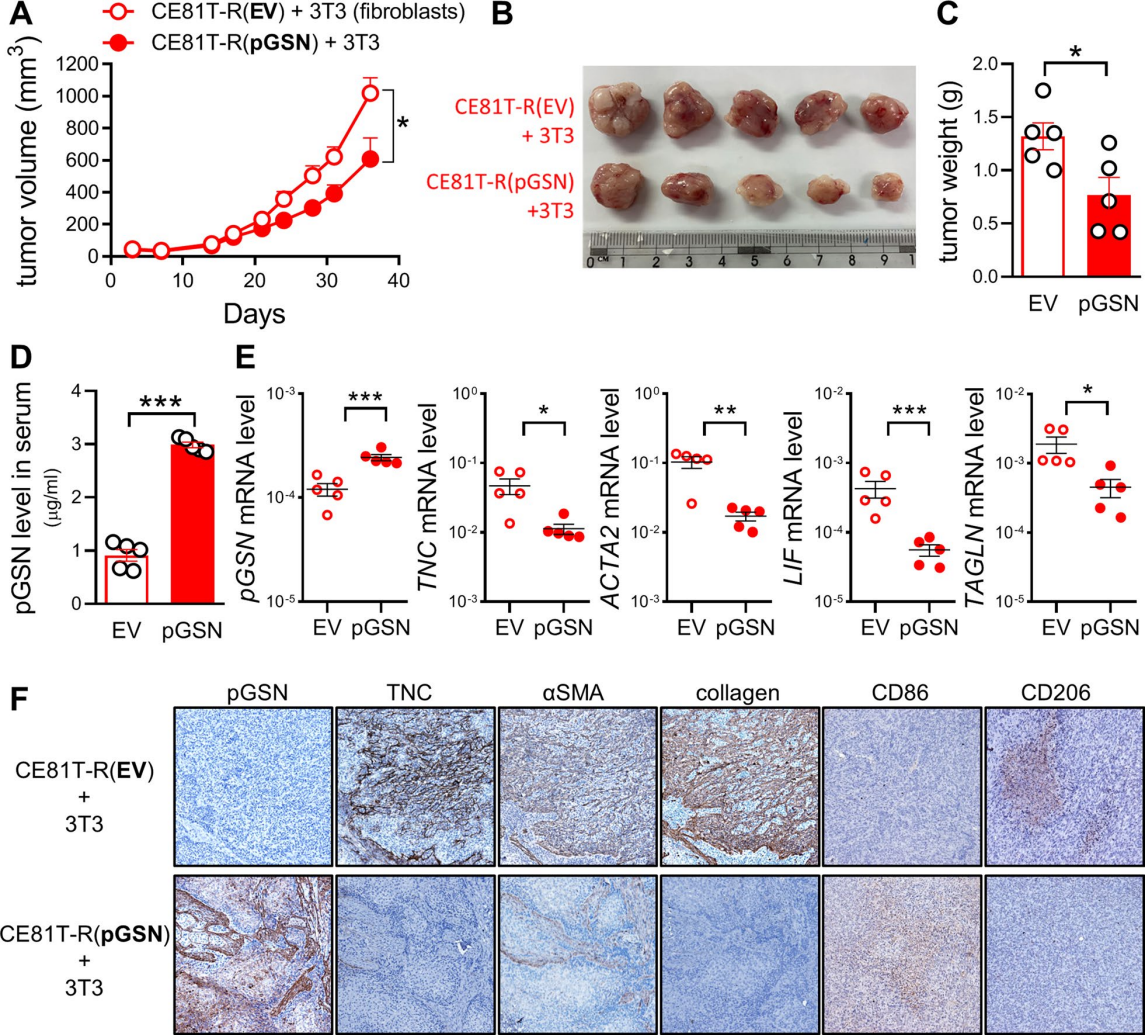
Overexpression of pGSN suppresses tumor growth by reducing TNC expression and CAF activation in vivo. **A** Tumor growth of xenografts co-transplanted with CE81T-R (EV or pGSN overexpression) and fibroblasts 3T3 cells. **B** and **C** Tumor size (**B**) and tumor weight (**C**) were measured at the end of the experiment. **D** ELISA analysis of circulating pGSN in mice at the end of the experiment. **E,** mRNA expressions of *pGSN*, *TNC*, and CAF markers (*ACTA2*, *LIF*, and *TAGLN*) were determined by RT-qPCR analysis. **F** IHC staining of pGSN, TNC, αSMA, collagen, CD86 M1 and CD206 M2 macrophage markers in tumors. Data represents mean ± s.e.m. ns: non-significant; *p < 0.05; **p < 0.01; ***p < 0.001

Next, to validate our observation that cancer-derived pGSN attenuates the activation of fibroblast to CAF in cell-based studies, we established ESCC tumor xenografts by subcutaneous inoculation of 10:1 mixture of CE81T-R (cancer) and 3T3 (fibroblast) cells. The results showed that fibroblast promoted the tumor growth and size as compared to the cancer xenograft model (Figs. 7A and S7A), and pGSN overexpression in cancer cells significantly increased the circulating pGSN level but reduced tumor growth, size, and weight (Fig. 7A–D). Additionally, body weight (Fig. S7F) and blood biochemistry analysis (Fig. S7G) showed no obvious difference between EV control and pGSN-overexpressing groups.

Moreover, we performed RT-qPCR analysis of the tumor tissues (Figs. 7E and S7H). The results revealed that mRNA expression of oncogenic TNC was reduced in the pGSN-overexpressing tumors, suggesting that pGSN disrupts the TNC-integrin positive feedback loop and decreases TNC transcription in vivo. In addition, mRNA expression of CAF markers (*ACTA2, TAGLN,* and *LIF*) was also down-regulated in the pGSN-overexpressing tumors. Of note, IHC analysis demonstrated a consistent result that the expression of oncogenic TNC and CAF markers (αSMA and collagen I), as well as M2-like pro-tumoral macrophages (stained as CD206 +) were notably decreased in the pGSN-overexpressing tumor tissues (Fig. 7F and S7I), supporting the notion that pGSN induced anti-tumor and pro-inflammatory immunomodulation. Altogether, these data suggested that tumor-suppressive pGSN could reduce TNC expression due to the competition between pGSN and TNC for integrin binding. CAF activation and M2-predominant pro-tumor microenvironment were also suppressed in vivo. Therefore, pGSN is a potential target for anti-ESCC treatment.

## Discussion

**Fig. 8:**
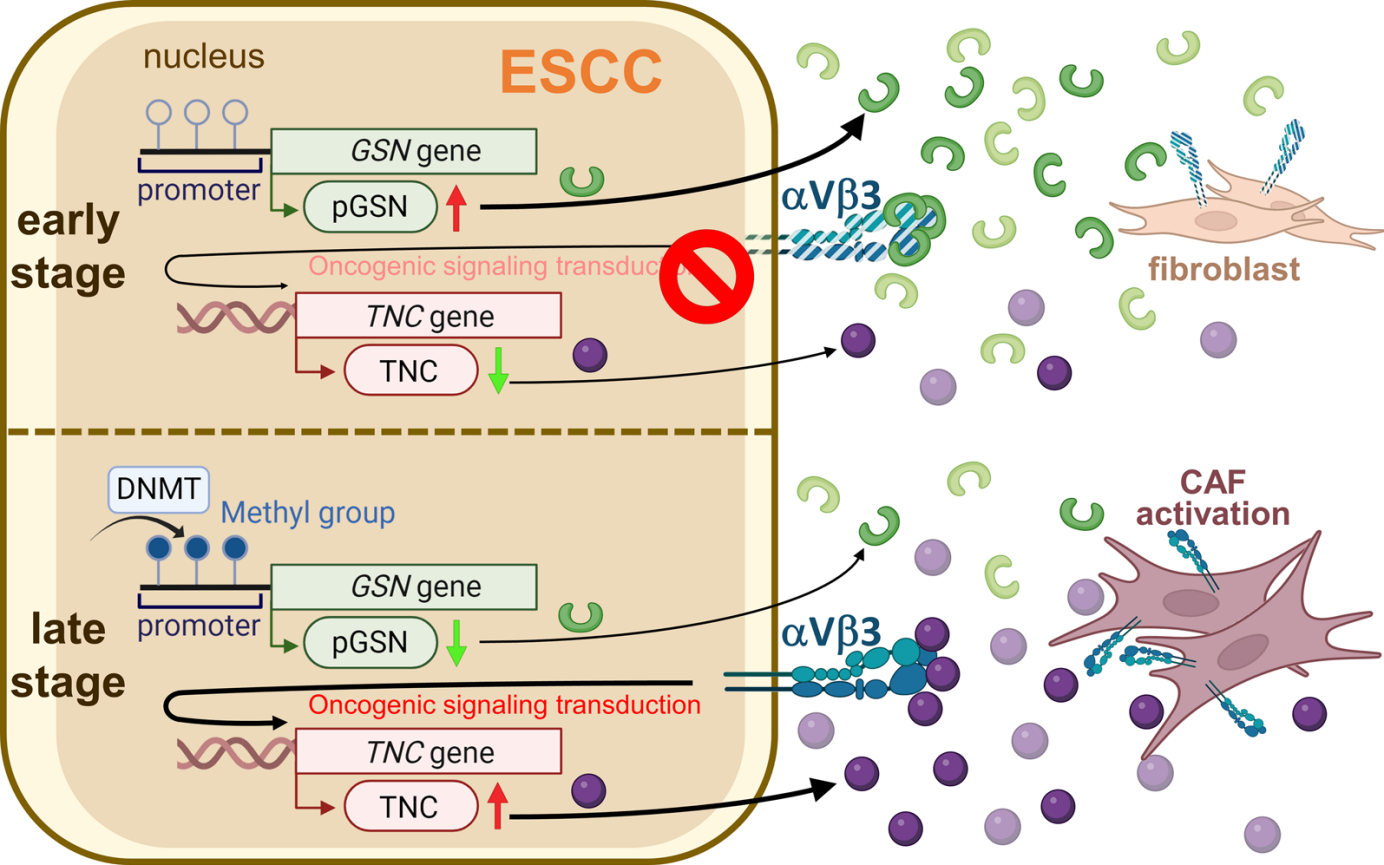
Schematic model of pGSN deficient fostering a fibrotic tumor environment of ESCC. In early-stage ESCC patients, extracellular pGSN competes with oncogenic TNC for binding to integrin αvβ3, reducing its stability and suppressing oncogenic signaling. During ESCC tumor progression, *GSN* gene methylation causes decreased secretion of pGSN, leading to integrin αvβ3 dysregulation, oncogenic TNC activation, and CAF formation

In this study, we discovered that the circulating level of pGSN is distinctly decreased during ESCC tumor progression. Patients with poor CCRT response have lower pGSN levels as compared to patients with good response, and Kaplan–Meier survival analysis indicated that low pGSN levels in patients was highly correlated with poor survival. In addition, cell-based studies showed that pGSN overexpression in cancer cells reduced proliferation, migration, invasion, and drug resistance, revealing that pGSN plays a tumor-suppressive role in ESCC. Interestingly, methylation-specific PCR analysis revealed that the decrease in pGSN is partly attributed to *GSN* promoter hypermethylation. Remarkably, IP and cycloheximide chase analyses demonstrated that pGSN not only competes with oncogenic TNC expression for binding to integrin αvβ3, but also decreased protein stability of integrin αvβ3, leading to suppression of oncogenic signaling transduction. Furthermore, pGSN-overexpressing cancer cell-derived condition media attenuates the conversion of fibroblasts to CAFs. Importantly, the cancer cell and fibroblast co-transplantation tumor model confirmed that pGSN overexpression reduces TNC expression and attenuates CAF activation, thereby suppressing tumor growth (Fig. 8).

Findings of recent comprehensive database analyses showed that *GSN* promoter is hypermethylated in many cancers, including head and neck squamous carcinoma and lung squamous cell carcinoma [32, 33]. Here, our clinical and cell-based studies confirmed that *GSN* is hypermethylated, and pGSN expression is significantly inversely correlates with protein expression of DNMTs in ESCC. Of note, previous investigations found that patients with poor CCRT response showed less infiltration of anti-cancer immune cells [34]. Importantly, *GSN* expression was reported to be positively associated with anti-tumor immunity [33]. Therefore, we suggested that decreased pGSN due to DNA methylation in ESCC may attenuate anti-tumor immunity and thus cause therapeutic resistance. Interestingly, tumor-associated M2-like macrophages could facilitate *GSN* methylation in gastric cancer cells to promote cancer progression [35]. Therefore, DNA methylation of tumor suppressor *GSN* gene could be regulated by cancer cells per se or tumor-infiltrating immune cells. Further study is required to comprehensively understand the molecular mechanism by which low pGSN expression reduces anti-cancer immunity and thus causes therapeutic resistance in ESCC.

In addition to stromal immune cells, CAFs constitute the predominant stromal cellular component in tumor microenvironment. Of note, fibroblasts isolated from human esophagus tissues were reported to promote ESCC progression in vitro and in vivo [36]. Here, we discovered that pGSN decreased the differentiation of fibroblasts toward CAFs, and IHC analysis showed low pGSN expression/high expression of CAF markers in late-stage ESCC patients. Hence, we suggested that decreased pGSN-induced ESCC progression could be partly attributed to the activation of CAFs. Interestingly, the isolated esophagus fibroblasts were also demonstrated to attenuate the anti-cancer effects of lapatinib treatment on ESCC [36], which agrees with the clinicopathological findings that ESCC recurrence after CCRT treatment is closely associated with stromal fibrosis [37]. Therefore, the decreased pGSN-induced CAF activation may further result in CCRT resistance, and re-expression of pGSN or CAF inactivation could be potential strategies to overcome CCRT resistance in ESCC.

As a secreted plasma protein, pGSN is well recognized as an extracellular actin scavenger [14]. pGSN was also found to interact with fibronectin; however, the mechanisms underlying the ECM evolution modulated by pGSN remains unclear. In this study, we discovered that pGSN competes with TNC for αvβ3 integrin binding and thus disrupts oncogenic signaling transduction in cancer cells as well as fibroblasts. Together with cell behavior observation, our data identified pGSN as a tumor suppressor in ESCC. The key events for pGSN tumor-suppressing action and TNC pro-tumorigenic effect in ESCCs involved in the integrin-dependent signaling pathways are worthy of further investigation. Nevertheless, several reports showed that pGSN demonstrated oncogenic properties in cervical carcinoma and ovarian cancer [38, 39]. Thus, we suggested that whether pGSN plays an oncogenic or tumor-suppressive role is cancer-type dependent and could be correlated with the physiologic state of the tumor microenvironment. Remarkably, an FDA-approved nonsteroidal anti-inflammatory drug, indomethacin, was demonstrated to significantly inhibit ESCC patient‐derived xenograft tumor growth and recurrence by targeting integrin αvβ3 for degradation [30]. Here, we found that pGSN overexpression decreases the stability of integrin αvβ3. Therefore, decreased pGSN expression during tumor progression could be a promising biomarker for indomethacin treatment of ESCC.

## Conclusions

This study identifies a novel integrin binding competition between pGSN and TNC, and *GSN* gene methylation during ESCC tumor progression causes decreased secretion of pGSN, leading to integrin αvβ3 dysregulation, oncogenic TNC activation, and CAF formation. These findings highlight the role of pGSN in therapeutic resistance and fibrotic tumor environment of ESCC, and thus pGSN appears to be a potential therapeutic target and biomarker.

## Supplementary Information

Supplementary file 1.

## Data Availability

All data used during the current study available from the corresponding author on reasonable request.

## Abbreviations

αSMA: α Smooth muscle actin
5-aza: 5-Aza-2’-deoxycitidine
CAF: Cancer-associated fibroblast
CCRT: Concomitant chemo-radiation therapy
cGSN: Cytoplasmic gelsolin
CHX: Cycloheximide
CM: Conditioned media
DNMTs: DNA methyltransferases
ECM: Extracellular matrix
ELISA: Enzyme-linked immunosorbent assay
ESCC: Esophageal squamous cell carcinoma
EV: Empty vector
FAP: Fibroblast activation protein
IF: Immunofluorescence
IHC: Immunohistochemistry
IP: Immunoprecipitation
M: Methylated
MSP: Methylation-specific PCR
OE: Overexpression
P: Parental
pGSN: Plasma gelsolin
R: Resistant
RT-qPCR: Quantitative reverse transcription polymerase chain reaction
TNC: Tenascin-C
U: Unmethylated
