# Supplementary material for: Decreased plasma gelsolin fosters a fibrotic tumor microenvironment and promotes chemoradiotherapy resistance in esophageal squamous cell carcinoma
Source: J Biomed Sci. 2024 Sep 11;31:90. doi: 10.1186/s12929-024-01078-7 (PMC11389350; doi:10.1186/s12929-024-01078-7)
Supplement: Supplementary file 1 [file 12929_2024_1078_MOESM1_ESM.pdf]

Additional file 1: Supplementary Information

Decreased plasma gelsolin fosters a fibrotic tumor microenvironment and promotes chemoradiotherapy resistance in esophageal squamous cell carcinoma

Chih-Hsiung Hsieh, Pei-Shiuan Ho, Wen-Lun Wang, Fu-Hsuan Shih, Chen-Tai Hong, Pei-Wen Wang, Dar-Bin Shieh, Wei-Lun Chang, and Yi-Ching Wang

Supplementary Materials and Methods

Colony formation assay

Transwell migration and invasion assays

AlphaFold 3.0 prediction and PyMOL analysis

Supplementary Figures and Tables

Supplementary Figure S1 is related to Figure 1.

Supplementary Figure S2 is related to Figure 2.

Supplementary Figure S3 is related to Figure 3.

Supplementary Figure S4 is related to Figure 4.

Supplementary Figure S5 is related to Figure 5.

Supplementary Figure S6 is related to Figure 6.

Supplementary Figure S7 is related to Figure 7.

Supplementary Table S1 is related to Materials and Methods.

Supplementary Table S2 is related to Materials and Methods.

Supplementary Table S3 is related to Materials and Methods.

Supplementary Table S4 is related to clinicopathological parameter analysis.

Supplementary Materials and Methods

Colony formation assay

Transfected ESCC cells were seeded at low density (500 cells per well for KYSE510-pair and 3,000 cells per well for CE81T-pair or CE48T-pair) in 6-well plates for 9~14 days. The cells were washed twice with PBS and fixed at room temperature, followed by staining with crystal violet for 30 min. Cell colonies were counted and analyzed using ImageJ software.

Transwell migration and invasion assays

For the invasion assay, the transwell membranes (Falcon, Franklin Lakes, NJ, USA) were pre-coated with Matrigel (Corning, New York, NY, USA) 1 day before the experiment. For both migration and invasion assays, 5×10^5 cells were seeded onto the upper chamber of the transwell with 1 mL serum-free medium, and the lower chamber was filled with 2 mL medium containing 20% fetal bovine serum (Gibco, Waltham, MA, USA), followed by incubation at 37°C for 20 hr. Cells invading the reverse side of the transwell membrane were fixed with 1% formaldehyde and then stained with 0.1% crystal violet at room temperature. Cell images were randomly photographed using Olympus CKX53 and analyzed using ImageJ software.

AlphaFold 3.0 prediction and PyMOL analysis

The human amino acid sequences of plasma gesolin (Uniprot: P06396), tenascin-C (Uniprot: P24821), integrin alpha-v (Uniprot: P06756), and integrin beta-3 (Uniprot: P05106) were submitted to Alphafold3 (https://alphafoldserver.com/) for the prediction of candidate 3D protein-protein interaction models. The predicted model files and potential interaction sites were visualized in PyMOL 3.0, and the 3D structures and complexes were subsequently exported.

Supplementary Figures and Tables

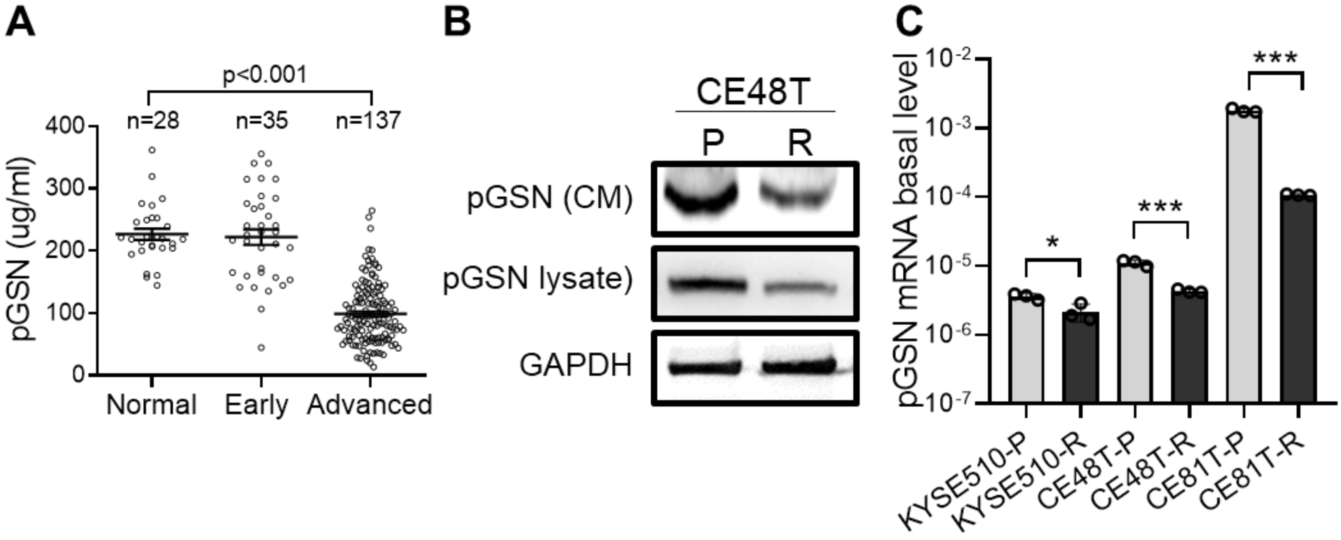

Supplementary Figure S1 is related to Figure 1.

Clinical and cell-based analyses of pGSN expression. A, ELISA analysis of circulating pGSN in ESCC patients (N=172) stratified by different stages. A total of 28 normal individuals are included as a control group. **B,** Basal levels of pGSN expression in CE48T cell lysate and CM of the resistant (R) or parental (P) cells were examined by Western blot analysis. GAPDH was used as an internal control. **C,** Basal levels of *pGSN* mRNA expression in ESCC cell lines were determined by RT-qPCR analysis. Data represents mean ± s.e.m. ns: non-significant; *, $p<0.05$; **, $p<0.01$; ***, $p<0.001$.

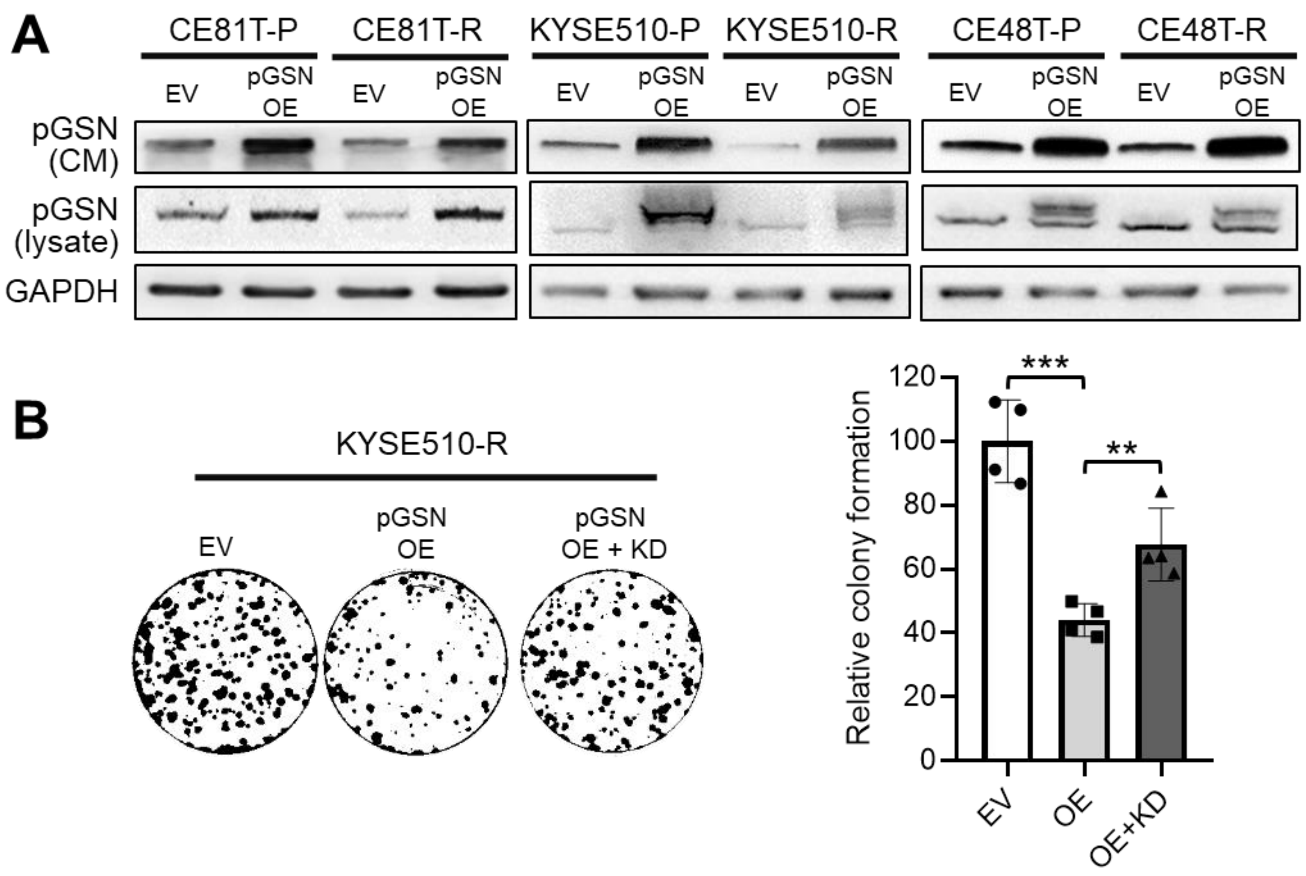

Supplementary Figure S2 is related to Figure 2.

Examination of the effect of pGSN overexpression on the oncogenicity of ESCC cells *in vitro*. A, Western blot analysis of pGSN expression after pGSN overexpression in ESCC cells. **B,** Colony formation assay of KYSE510-R cells with overexpression (OE) and/or knockdown (KD) of pGSN. The colonies were stained on day 8 after seeding. Data represents mean ± s.e.m. **, p<0.01; ***, p<0.001.

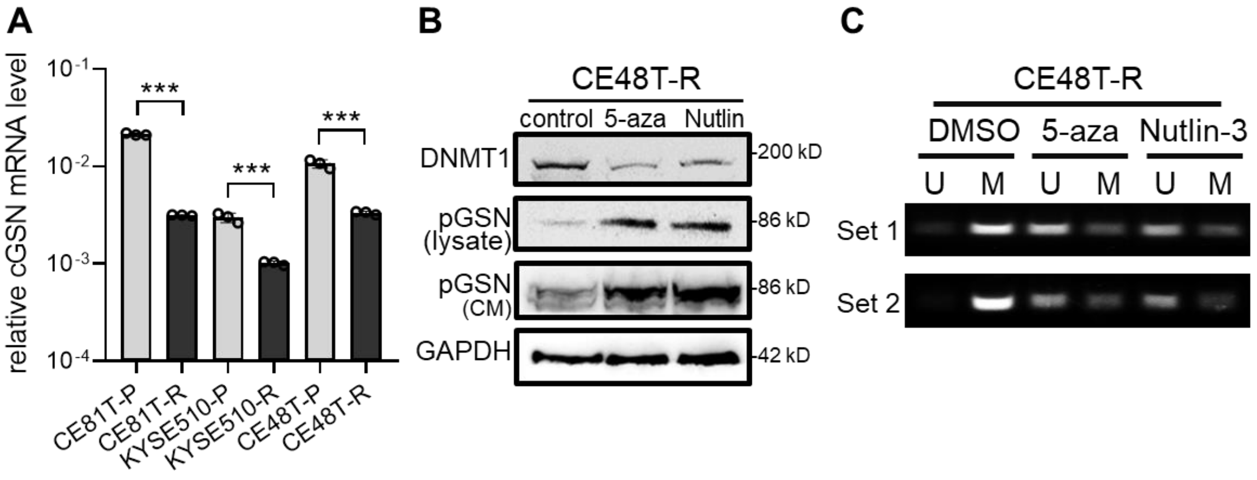

Supplementary Figure S3 is related to Figure 3.

Investigation of *GSN* promoter hypermethylation. A, Basal levels of *cGSN* mRNA expression in ESCC cell lines were determined by RT-qPCR analysis. **B,** Immunoblotting of DNMT1 and pGSN in CE48T-R cells treated with demethylation agents 5-aza or Nutlin-3. GAPDH was used as an internal control. **C,** MSP results demonstrated that demethylation agents (5-aza or Nutlin-3) reduced the methylation of *GSN* promoter. Data represents mean ± s.e.m. ns: non-significant; $*p < 0.05$; $**p < 0.01$; $***p < 0.001$.

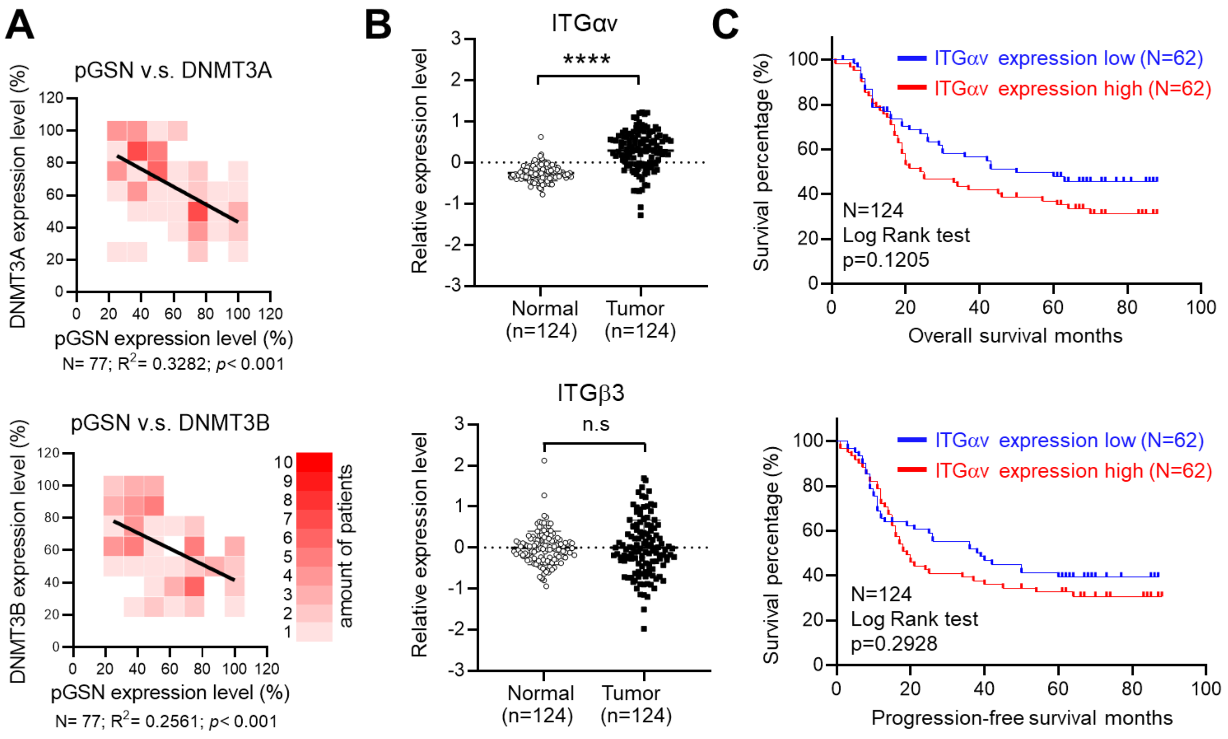

Supplementary Figure S4 is related to Figure 4.

pGSN inversely correlates with the expression of DNMT3A, DNMT3B, and integrin αvβ3. A, The correlation analysis of pGSN with DNMT3A (*upper*) and DNMT3B (*lower*) expression in 77 patient tumor tissues. **B,** Protein levels of integrin αvβ3 (ITGαv and ITGβ3) in the ESCC proteomic mass spectrum dataset (24). **C,** Kaplan–Meier plots of overall survival (*upper*) (**D**) and progression-free survival (*lower*) of ITGαv.

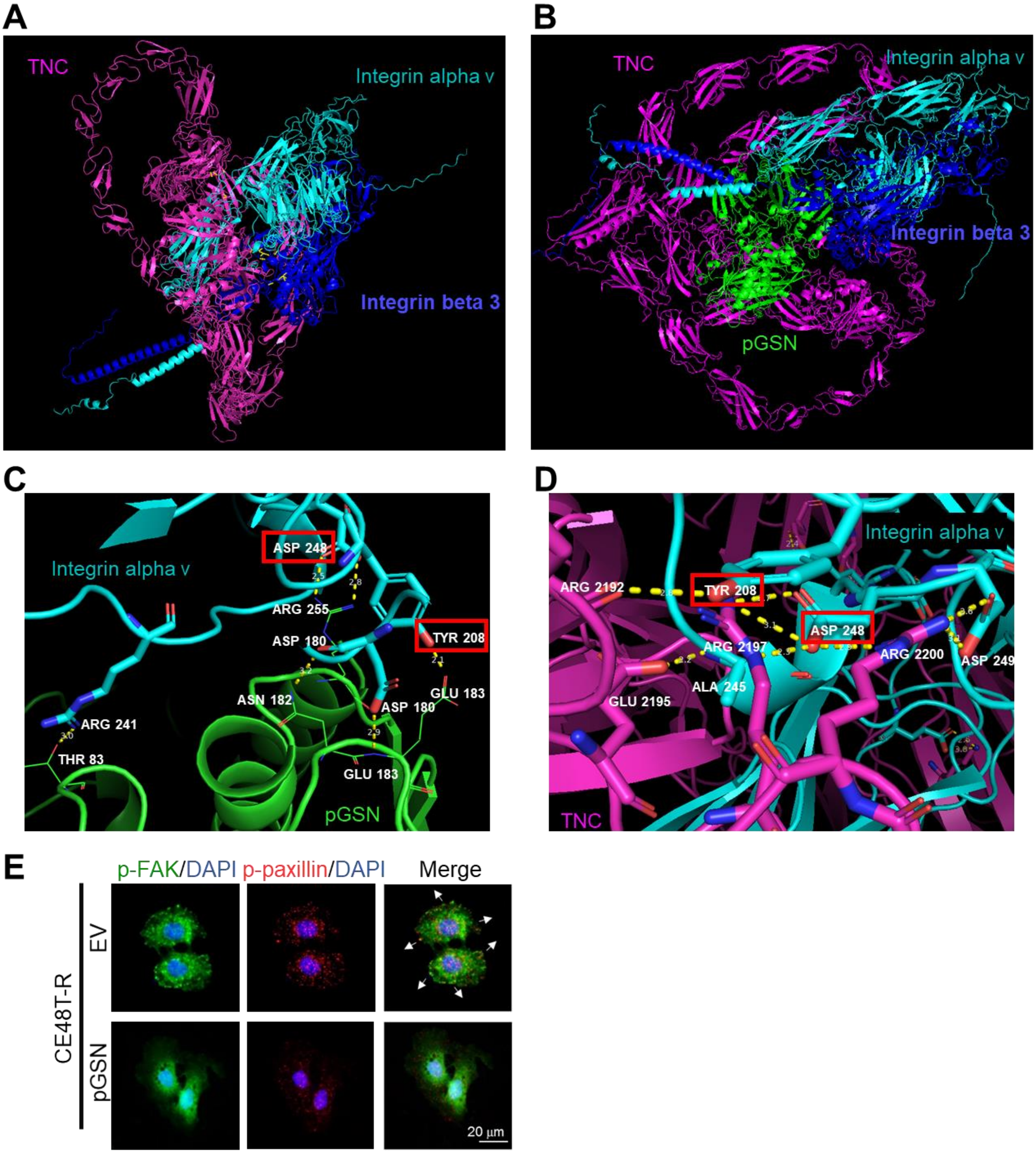

Supplementary Figure S5 is related to Figure 5.

The interaction modes among TNC, integrin αvβ3, and pGSN, as well as integrin downstream signaling. A and B, The predicted heteromer complexes between TNC (purple), integrin αvβ3 (light and dark blue), and pGSN (green). **C and D,** The predicted interaction amino acids between the integrin αv (light blue) and the pGSN (C) as well as integrin αv (light blue) and the TNC (purple) (D) by AlphaFold 3.0. **F,** pGSN inhibits integrin downstream signaling. Integrin downstream signaling p-FAK and p-paxillin in CE48T-R cells were examined by immunofluorescence staining.

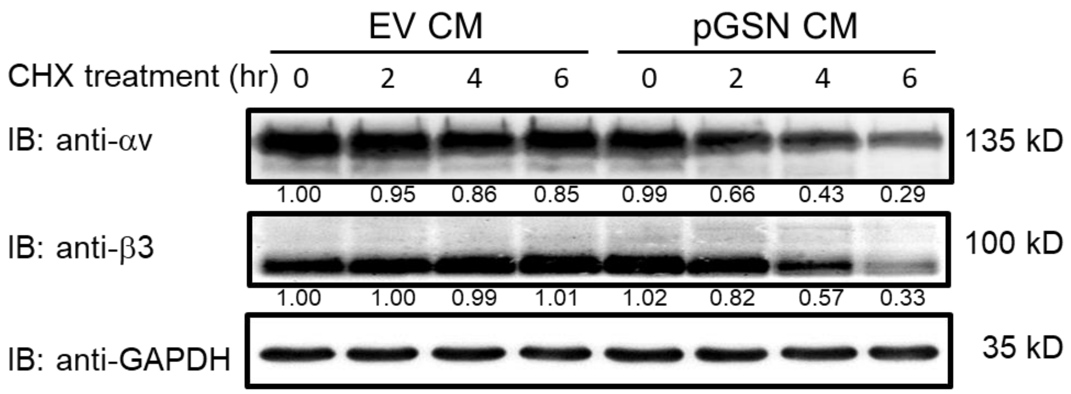

Supplementary Figure S6 is related to Figure 6.

pGSN reduces integrin αvβ3 protein stability. Western blot analysis of CHX chase assay to investigate the protein stability of integrin αvβ3 in 3T3 fibroblast cells. GAPDH was used as an internal control.

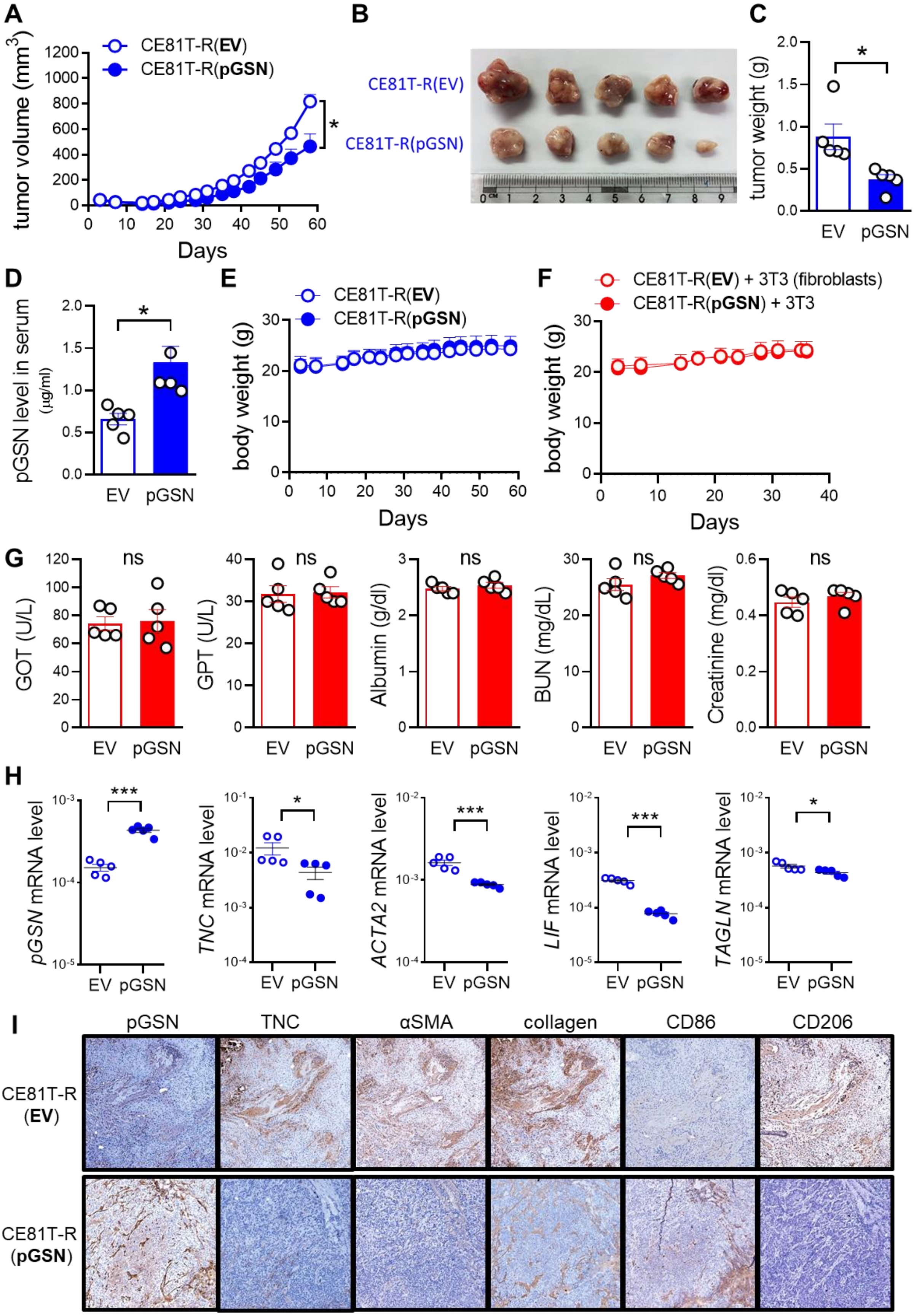

Supplementary Figure S7 is related to Figure 7.

pGSN-induced anti-tumor effects on ESCC and fibroblasts *in vivo*. A, Tumor growth of xenografts

transplanted with CE81T-R (EV or pGSN overexpression) cells. **B** and **C,** Tumor size (**B**) and tumor weight (**C**) were measured at the end of the experiment. **D,** ELISA analysis of circulating pGSN in mice at the end of the experiment. **E** and **F,** Body weight of mice transplanted with CE81T-R (**E**) or co-transplanted with CE81T-R and 3T3 cells (**F**) was recorded during the animal experiment. **G,** The blood biochemistry analysis of mice co-transplanted with CE81T-R and 3T3 cells. **H,** mRNA expressions of *pGSN*, *TNC*, and CAF markers (*ACTA2*, *LIF*, and *TAGLN*) in mice transplanted with CE81T-R cells were determined by RT-qPCR analysis. **I,** IHC staining of pGSN, TNC, αSMA, collagen, CD86 M1 and CD206 M2 macrophage markers in tumors. Data represents mean ± s.e.m. ns: non-significant; *$p < 0.05$; **$p < 0.01$; ***$p < 0.001$.

Supplementary Table S1. The plasmids and their characteristics used in the study.

Plasmid	Target	Insert (bp)	Function
pcDNA3.1/myc-His A	None	-[a]	Vector control
pcDNA3.1/myc-His A-pGSN	*GSN isoform a*	1,242	Overexpression

[a] The plasmid is used as a backbone vector therefore there is no insert DNA fragment.

Supplementary Table S2. The antibodies and their reaction conditions used in the study.

Target	MW (kDa)	Raised in	Application[a]	Dilution	Source	Catalog No.
GSN	80, 85	Mouse	WB<br>IHC	1:1000<br>1:100	Abcam	ab11081
pGSN	85	Rabbit	WB<br>IHC	1:1000<br>1:100	Abcam	ab75832
DNMT1	183	Rabbit	WB<br>IHC	1:1000<br>1:500	GeneTex	GTX116011
DNMT3A	102	Rabbit	WB<br>IHC	1:1000<br>1:100	GeneTex	GTX129125
DNMT3B	96	Rabbit	WB<br>IHC	1:1000<br>1:100	Cell Signaling	#2161
TNC	220	Rabbit	WB<br>IHC	1:500<br>1:100	Santa Cruz	SC-20932
α-SMA	42	Rabbit	IHC	1:250	GeneTex	GTX100034
Collagen I	139	Rabbit	IHC	1:100	Abcam	ab34710
Normal mouse IgG	-	Mouse	IP	1:5000	Sigma-Aldrich	12-371
αVβ3 integrin	-	Mouse	IP	1:500	Abcam	ab190147
αV integrin	116	Rabbit	WB	1:1000	GeneTex	GTX100789
β3 integrin	87	Rabbit	WB	1:1000	GeneTex	GTX01182

p-FAK (y-397)	125	Rabbit	IF	1:200	Abcam	ab4803
p-paxillin (y-31)	68	Rabbit	IF	1:200	Abcam	ab32115
β-actin	42	Mouse	WB	1:2000	GeneTex	GTX26276
GAPDH	37	Mouse	WB	1:2000	Proteintech	60004-1-Ig

[a] WB: western blot; IHC: immunohistochemistry; IP: immunoprecipitation; IF: immunofluorescence.

Supplementary Table S3. The primers used in the study.

Protein (gene name)	Primer	Sequences (5'→ 3')	Application[a]	PCR size (bp)	Tm (°C)
pGSN promoter site 1 U primer	Forward	TgggAATTTAgATgTTTTTAAgATTT	MS-PCR	147	50
	Reverse	AACAACAAAAAACCCAATCAAC			
pGSN promoter site 1 M primer	Forward	TgggAATTTAgATgTTTTTAAgATTC	MS-PCR	147	55
	Reverse	AACgACAAAAAACCCAATCg			
pGSN promoter site 2 U primer	Forward	TTgggTTTgTTgTTgTTTgT	MS-PCR	151	50
	Reverse	CATCTAAATTCCCACCCAAA			
pGSN promoter site 2 M primer	Forward	AgTTgggTTCgTCgTCgTTC	MS-PCR	153	55
	Reverse	CATCTAAATTCCCACCCgAA			
pGSN promoter site 3 U primer	Forward	ggggTTATTTTAggAgTTgAgT	MS-PCR	113	50
	Reverse	CCCCCACCAAACAACATACAC			
pGSN promoter site 3 M primer	Forward	gggTTATTTTAggAgTTgAgCg	MS-PCR	114	55
	Reverse	CCCCCACCAAACAACATACg			
pGSN (*GSN isoform a*)	Forward	CTgCTTTgCgCgCTgTC	RT-qPCR	104	60
	Reverse	TgTTCCACCACCATgCTgTT			
cGSN (*GSN isoform b*)	Forward	AgCAgCCgCTgTCTCCAgT	RT-qPCR	125	60
	Reverse	CTAACAAggCTgTgCAgggTg			
Human α-SMA *(ACTA2)*	Forward	AgATCAAgATCATTgCCCC	RT-qPCR	116	60
	Reverse	TTCATCgTATTCTgTTTgC			

Human transgelin *(TAGLN)*	Forward	ggTggAgTggATCATAgTgC	RT-qPCR	154	60
	Reverse	ATgTCAgTCTTgATgACCCCA			
Human Leukemia inhibitory factor *(LIF)*	Forward	CTgTTggTTCTgCACTggAA	RT-qPCR	243	60
	Reverse	CCCCTgggCTgTgTAATAgA			
Human β-actin *(ACTB)*	Forward	ggCggCACCACCATgTACCCT	RT-qPCR	180	60
	Reverse	AggggCCggACTCgTCATACT			
Mouse *α-SMA (ACTA2)*	Forward	gCATCCgACACTgCTgACAgA	RT-qPCR	149	60
	Reverse	CAgTTgTACgTCCAgAggCATA			
Mouse transgelin *(TAGLN)*	Forward	ACCTCTATgAAggTAAggATATggC	RT-qPCR	125	60
	Reverse	CTgggCTTTCTTCATAAACCAgTTg			
Mouse Leukemia inhibitory factor *(LIF)*	Forward	TCAACTggCACAgCTCAATggC	RT-qPCR	119	60
	Reverse	ggAAgTCTgTCATgTTAggCGC			
Mouse β-actin *(ACTB)*	Forward	ggCggCACCACCATgTACCCT	RT-qPCR	180	60
	Reverse	AggggCCggACTCgTCATACT			

[a.] MS-PCR: Methylation-specific polymerase chain reaction; RT-qPCR: Quantitative reverse-transcriptase polymerase chain reaction.

Supplementary Table S4. The correlation of circulating level of pGSN with clinicopathological parameters of ESCC patients.

Parameters		Patients	pGSN expression level		*p* value[b]
		140[a]	high (N=66, 47%)	Low (N=74, 53%)	
Age	<55	70	31 (44.3%)	39 (55.7%)	0.498
	≥55	70	35 (50%)	35 (50%)	
Gender	male	133	62 (46.6%)	71 (53.4%)	0.587
	female	7	4 (57.1%)	3 (42.9%)	
Alcohol	no	15	5 (33.3%)	10 (66.7%)	0.257
	yes	125	61 (48.8%)	64 (51.2%)	
Smoker	no	16	7 (43.8%)	9 (56.2)	0.773
	yes	124	59 (47.6%)	65 (52.4%)	
Stage	I, II, III	45	34 (75.6%)	11 (24.4%)	**<0.001**
	IV	95	32 (33.7%)	63 (66.3%)	
State	survival	50	22 (44%)	28 (56%)	**0.044**
	mortality	84	32 (38.1%)	52 (61.9%)	

[a] The patients with complete clinical pathological information were included for the correlation analysis.

[b] The data was analyzed by Pearson $\chi 2$ test with significant P values in bold.
